# Trophoblast cell surface antigen-2 phosphorylation triggered by binding of galectin-3 drives metastasis through down-regulation of E-cadherin

**DOI:** 10.1016/j.jbc.2023.104971

**Published:** 2023-06-27

**Authors:** Shungo Iwamoto, Yugo Mori, Tomoko Yamashita, Kazuki Ojima, Kaoru Akita, Shingo Togano, Shuhei Kushiyama, Masakazu Yashiro, Yuki Yatera, Tomoko Yamaguchi, Akane Komiyama, Yuki Sago, Naoki Itano, Hiroshi Nakada

**Affiliations:** 1 Department of Molecular Biosciences, Faculty of Life Sciences, Kyoto Sangyo University, Kyoto, Japan; 2 Department of Molecular Oncology and Therapeutics, Osaka Metropolitan University Graduate School of Medicine, Osaka, Japan

**Keywords:** Trop-2, metastasis, E-cadherin, ZEB1, cell signaling

## Abstract

The expression of trophoblast cell surface antigen-2 (Trop-2) is enhanced in many tumor tissues and is correlated with increased malignancy and poor survival of patients with cancer. Previously, we demonstrated that the Ser-322 residue of Trop-2 is phosphorylated by protein kinase Cα (PKCα) and PKCδ. Here, we demonstrate that phosphomimetic Trop-2 expressing cells have markedly decreased E-cadherin mRNA and protein levels. Consistently, mRNA and protein of the E-cadherin–repressing transcription factors zinc finger E-Box binding homeobox 1 (ZEB1) were elevated, suggesting transcriptional regulation of E-cadherin expression. The binding of galectin-3 to Trop-2 enhanced the phosphorylation and subsequent cleavage of Trop-2, followed by intracellular signaling by the resultant C-terminal fragment. Binding of β-catenin/transcription factor 4 (TCF4) along with the C-terminal fragment of Trop-2 to the *ZEB1* promoter upregulated ZEB1 expression. Of note, siRNA-mediated knockdown of β-catenin and TCF4 increased the expression of E-cadherin through ZEB1 downregulation. Knockdown of Trop-2 in MCF-7 cells and DU145 cells resulted in downregulation of ZEB1 and subsequent upregulation of E-cadherin. Furthermore, wild-type and phosphomimetic Trop-2 but not phosphorylation-blocked Trop-2 were detected in the liver and/or lung of some nude mice bearing primary tumors inoculated intraperitoneally or subcutaneously with wild-type or mutated Trop-2 expressing cells, suggesting that Trop-2 phosphorylation, plays an important role in tumor cell mobility *in vivo*, too. Together with our previous finding of Trop-2 dependent regulation of claudin-7, we suggest that the Trop-2–mediated cascade involves concurrent derangement of both tight and adherence junctions, which may drive metastasis of epithelial tumor cells.

At the initial stage of epithelial carcinogenesis, loss of organized cell–cell adhesion is a prerequisite to allow the mobility of malignant cells. Epithelial–mesenchymal transition (EMT) is considered to be an essential step to drive metastasis, in which epithelial tumor cells gradually lose the epithelial phenotype and then exhibit a mesenchymal phenotype (1, 2). Down-modulation of E-cadherin plays a major causative role in EMT (3, 4).

ZEB (5), Snail, and Twist (6) are major transcriptional factors that repress the expression of E-cadherin. These transcriptional factors, which are upregulated by signaling mediated by a variety of growth factors such as TGF-β and HGF, induce a transcriptional program to turn on EMT. EMT is promoted by sustained signaling by activated growth factor receptors, such as EGFR, TGFR, and HGFR (1). However, the mechanism by which growth factors approach and ligate to their receptors in the premalignant phase before dissemination of epithelial cells remains unknown. Although in the polarized epithelial tissues growth factors are secreted to the apical side, their receptors are localized basolaterally, which prevents the ligation of growth factors to their receptors (7). When the respiratory epithelium is wounded, the polarity of epithelial cells is lost, which allows a heregulin secreted to the apical side to bind to basolaterally localized HER2/ErgB2, leading to cell proliferation (8). Although this mechanism works for wound healing, a similar mechanism may be involved in the initiation of EMT.

From this spatial–temporal view of tumor progression, we speculated that early alteration in the epithelial sheet structure may be a prerequisite to lead to growth factor-inducing EMT.

Trop-2 is a highly glycosylated membrane protein with a molecular mass of 36 kDa and is highly expressed in a variety of epithelial cancer cells (9, 10). Trop-2 was originally identified on normal and malignant trophoblast cells (11, 12) and belongs to the family of tumor-associated calcium signal transducers (TACSTD) (9). Many reports demonstrated that enhanced expression of Trop-2 in tumor tissues is correlated with tumor malignancy (9, 13, 14). Thus, Trop-2 has been identified as an oncogene leading to invasiveness and tumorigenesis (15). Activation of the Trop-2–mediated ERK pathway leads to upregulation of invasiveness and metastasis through enhanced expression of MMP2 (16). The epithelial cell adhesion molecule (EpCAM) gene is another highly conserved member of the TACSTD gene family. They exhibit 49% sequence homology (17). Elevated expression of EpCAM is also frequently observed in many types of carcinomas (18). On the other hand, it has been reported that loss of Trop-2 promotes carcinogenesis and EMT in squamous cell carcinomas (19) and that a mutation of the Trop-2 gene impairs the function of tight junctions through decreased expression and altered subcellular localization of tight junction proteins claudin-1 and claudin-7 in gelatinous drop-like corneal dystrophy (GDLD) corneas (20, 21). In EpCAM knockout mice, the barrier function of the intestinal epithelium is impaired (22). These individual studies were not comprehensive. Thus, the function of Trop-2 remains obscure.

In a previous paper (23), we demonstrated that the Ser322 residue of Trop-2 was phosphorylated by PKCα and PKCδ. Trop-2 phosphorylation-blocked cells were less motile compared with wild-type and phosphomimetic Trop-2 expressing cells. In addition, up- and down-modulation of Trop-2 phosphorylation by PKC activation and inhibition resulted in up- and down-modulation of cell motility, respectively, suggesting that the phosphorylation of Trop-2 is critical for the regulation of cell motility. Actually, phosphorylation of Trop-2 led to down-modulation and mislocalization of claudin-7, probably due to decreased interaction of Trop-2 with claudin-7. In this context, we found that the expression of E-cadherin was also regulated through Trop-2 phosphorylation.

## Results

### Expression of E-cadherin in wild-type and mutated Trop-2–expressing HCT116 cells

**Figure 1:**
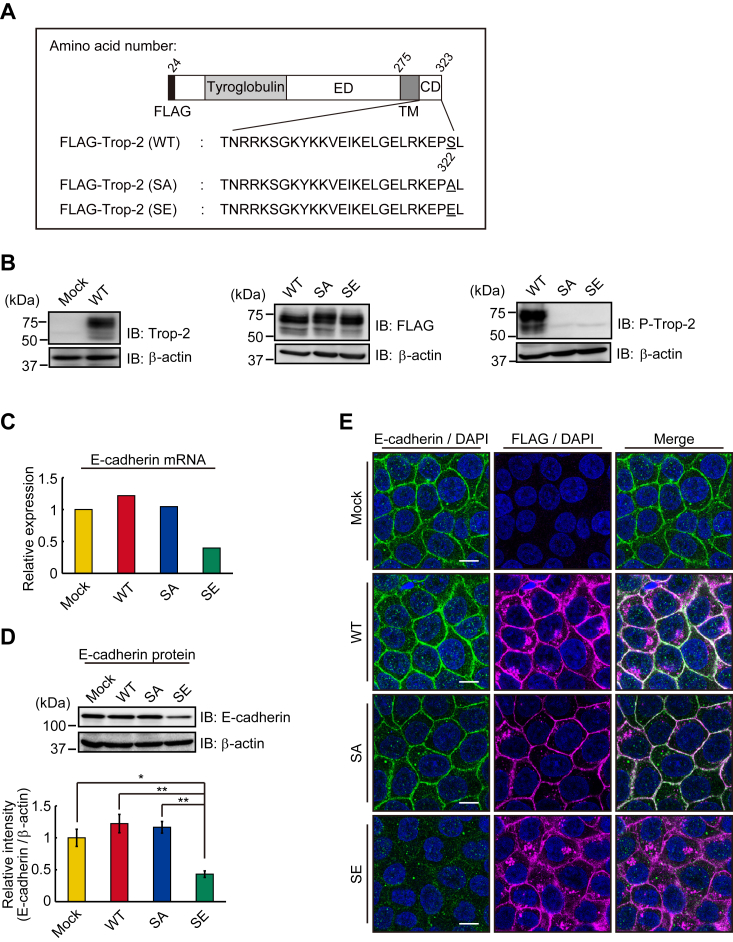
**Expression of E-cadherin in wild-type and mutated Trop-2 expressing HCT116 cells.***A*, schematic structures of wild-type and mutated Trop-2. *B*, (*left*) Lysates of HCT116/Mock (Mock) cells and HCT116 cells expressing wild-type Trop-2 (WT cells) were subjected to SDS-PAGE and immunoblotting, followed by detection with anti-Trop-2 antibodies. (*center*) Lysates of WT and HCT116 cells expressing mutated Trop-2 with Ala or Glu instead of Ser322 (SA, SE cells) were subjected to SDS-PAGE, followed by immunoblotting and detection with anti-FLAG-tag antibodies. (*right*) Three cell types (WT, SA, and SE cells) were treated with PMA (100 ng/ml) for 6 h, and their cell lysates were subjected to SDS-PAGE, followed by immunoblotting and detection with anti-phosphorylated Trop-2 antibodies. β-Actin served as a loading control. *C*, the levels of E-cadherin mRNA in the four cell types were determined by DNA microarray analysis (n = 1), and that in Mock cells was taken as 1. *D*, lysates prepared from the four cell types were subjected to SDS-PAGE, followed by immunoblotting, and detection with anti-E-cadherin antibodies. The intensities of the bands were determined. That in Mock cells was normalized as to β-actin, and the value was taken as 1. β-Actin served as a loading control (means ± S.E., n = 3, ∗*p* < 0.05, ∗∗*p* < 0.01). *E*, the distributions of E-cadherin (*green*) and FLAG-tagged Trop-2 (*magenta*) in the four cell types were observed immunochemically. Nuclei were stained with DAPI (*blue*). Scale bars, 10 μm.

In addition to wild-type Trop-2–expressing cells (HCT116/WT; WT cells), we prepared mutated Trop-2–expressing cells with Ala (HCT116/S322A; SA cells) and Glu (HCT116/S322E; SE cells) instead of Ser322 to block and mimic the phosphorylation, respectively, as described previously (Fig. 1*A*) (23). Confluent cells were dispersed with trypsin, seeded, and cultured for 24 h as described in the Experimental Procedures section. A lysate of HCT116/Mock cells was subjected to SDS-PAGE and immunoblotting, and endogenous Trop-2 was detected by using anti-Trop-2 antibodies. Its expression level was negligible compared with that of forcibly expressed Trop-2 in WT cells (Fig. 1*B*, *left*). Similar levels of Trop-2 expression in the three cell types were also confirmed on SDS-PAGE and immunoblotting by using anti-FLAG-tag antibodies (Fig. 1*B*, *center*), this being consistent with the flow cytometry analyses as described previously (23). We also prepared antibodies against the phosphorylated cytoplasmic peptide of Trop-2 and detected phosphorylated Trop-2 using this antibody (23). Since classical and novel PKCs are activated by a phorbol ester (PMA), lysates prepared from the three cell types treated with PMA for 6 h were subjected to SDS-PAGE and immunoblotting, followed by detection with anti-phosphorylated Trop-2 antibodies. Expectedly, phosphorylated Trop-2 was detected in WT cells but not in SA and SE cells (Fig. 1*B*, *right*).

In the previous article (23), we demonstrated that SA cells exhibited significantly less motility than the other Trop-2 expressing cells *in vitro* and that the motility of WT cells was effectively reduced by treatment with PKC inhibitor Gö6983. In relation to cell motility, it is also reported that Trop-2 is associated with EMT (24) and that high expression of Trop-2 and low expression of E-cadherin were observed in human breast cancer tissues, but a molecular correlation has not been elucidated (25).

Thus, we next compared the levels of E-cadherin in wild-type and mutated Trop-2 expressing HCT116 cells. Expectedly, E-cadherin mRNA and protein dramatically decreased in SE cells compared to other cells, suggesting that expression of E-cadherin was down-modulated transcriptionally (Fig. 1, *C* and *D*).

Furthermore, the distributions of E-cadherin and Trop-2 in wild-type and mutated Trop-2 expressing HCT116 cells were investigated immunochemically (Fig. 1*E*). While E-cadherin in SA cells was confined at the cell membrane as well as in normal epithelial cells, a part of E-cadherin in WT cells was localized in the cytosol. A diffuse distribution was observed more prominently in SE cells. These observations suggest that Trop-2 may play a causative role in the level and localization of E-cadherin.

### Expression of transcriptional factors that repress E-cadherin transcription

**Figure 2:**
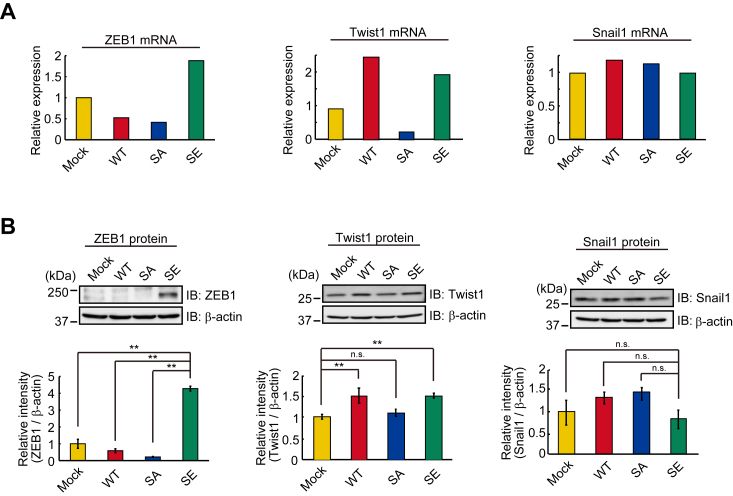
**Expression of transcriptional factors to down-modulate E-cadherin transcription.***A*, the levels of ZEB1, Twist1, and Snail1 mRNAs in the four cell types were determined by DNA microarray analysis (n = 1), and that in Mock cells was taken as 1. *B*, lysates prepared from the four cell types were subjected to SDS-PAGE and blotting. The blotted membrane was cut into three pieces containing proteins with high, intermediate, and low molecular masses, followed by detection of ZEB1, β-actin, and Snail1 proteins with each antibody, respectively. Because ZEB1, Snail1, and β-actin proteins were detected using the same blotted membrane, the β-actin image is shown as a loading control of both ZEB1 and Snail1 proteins. Separately, β-actin and Twist1 proteins were detected through the same procedure as described above. This β-actin image is shown as a loading control of the Twist1 protein. The intensities of the bands were determined. That in Mock cells was normalized as to β-actin, and the value was taken as 1. (means ± S.E., n = 3, ∗∗*p* < 0.01, n.s.: not significant).

It is well-known that E-cadherin is a hallmark of EMT and that EMT is driven by Snail, ZEB, and Twist transcriptional factors (5, 6). Thus, we analyzed the levels of mRNA and protein of these transcriptional factors in mock, wild-type, and mutated Trop-2 expressing cells by DNA microarray analyses (Fig. 2*A*) and immunoblotting (Fig. 2*B*). Similar levels of Snail1 mRNA and protein were expressed in these cells. ZEB1 mRNA was markedly increased in SE cells, and Twist1 mRNA was elevated in WT and SE cells. E-cadherin protein was decreased only in SE cells, suggesting that ZEB1 mainly regulates the expression of E-cadherin, but there may be two pathways to upregulate the transcription of ZEB1 and Twist1 through downstream events after Trop-2 phosphorylation. The reason why E-cadherin did not decrease in WT cells (Fig. 1, *C* and *D*) despite the up-regulation of Twist1 (Fig. 2, *A* and *B*) is discussed later.

Although Trop-2 is expressed broadly in cancers (14, 26, 27, 28, 29), little is known about Trop-2–mediated signaling. In addition, it remains to be determined whether or not Trop-2 phosphorylation is related to signaling.

### Enhanced cleavage of Trop-2 through its phosphorylation and accumulation of β-catenin in the nucleus

**Figure 3:**
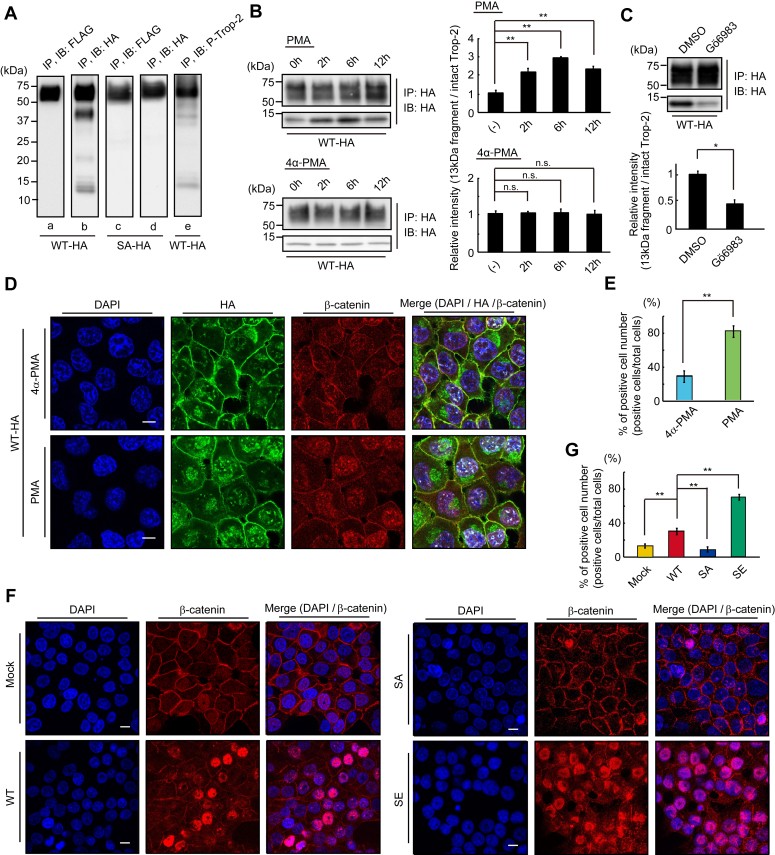
**Enhanced cleavage of Trop-2 through its phosphorylation and accumulation of β-catenin in the nucleus.***A*, FLAG- and HA-tagged Trop-2 were immunoprecipitated from lysates of HCT116/WT-HA (WT-HA) cells (lanes a, b) and HCT116/SA-HA (SA-HA) cells (lanes c, d), and then subjected to SDS-PAGE, followed by immunoblotting and detection with anti-FLAG-tag or HA-tag antibodies. HA-tagged Trop-2 was also immunoprecipitated from lysates of WT-HA cells and subjected to SDS and immunoblotting, followed by detection with anti-phosphorylated Trop-2 antibodies (lane e). *B*, HA-tagged Trop-2 was immunoprecipitated from lysates of WT-HA cells treated with PMA (100 ng/ml) or with 4α-PMA for the indicated times and then subjected to SDS-PAGE, followed by immunoblotting and detection with anti-HA-tag antibodies. The intact Trop-2 and intracellular fragment with a molecular mass of ∼13 kDa were demonstrated. The ratio of HA-tagged ∼13-kDa fragment to HA-tagged intact Trop-2 was calculated, and the value for the cells treated with PMA or 4α-PMA for 0 h was taken as 1 (means ± S.E., n = 3, ∗∗*p* < 0.01, n.s.: not significant). *C*, WT-HA cells were treated with Gö6983 (1 μM) or DMSO (control) for 2 h and subsequently with PMA (100 ng/ml) for an additional 6 h, and the intact molecule and ∼13-kDa fragment were detected as described above. The ratio of HA-tagged ∼13 kDa fragment to HA-tagged intact Trop-2 was calculated, and the value for the control experiment (DMSO) was taken as 1 (means ± S.E., n = 3, ∗*p* < 0.05). *D*, WT-HA cells were treated with PMA (100 ng/ml) or 4α-PMA (control) for 6 h and then immunostained with anti-HA-tag (*green*) and anti-β-catenin (*red*) antibodies. Nuclei were stained with DAPI (*blue*), and a representative photograph is shown. Scale bars, 10 μm. *E*, the nuclear transport of HA-tagged Trop-2 fragment and β-catenin was calculated by counting the white dots (β-catenin colocalized with the Trop-2 fragment) per five randomly selected fields in merged images in Figure 3*D*. The ratio (%) of white dot positive cells per total cells was calculated (means ± S.E., n = 5, ∗∗*p* < 0.01). *F*, β-catenin in the four cell types was immunostained with anti-β-catenin antibodies (*red*). Nuclei were stained with DAPI (*blue*), and a representative photograph is shown. Scale bars, 10 μm. *G*, the ratio (%) of nuclear β-catenin positive cells per total cells in five randomly selected fields was calculated and is shown as a histogram (means ± *S.E.*, n = 5, ∗∗*p* < 0.01).

It is reported that Trop-2 is cleaved through regulated intramembrane proteolysis (RIP), which is performed by the TNF-α-converting enzyme (TACE) and subsequently by γ-secretase, resulting in shedding of its extracellular domain and nuclear translocation of its intracellular peptide (30). In order to detect the intracellular peptide and investigate whether or not Trop-2 phosphorylation is related to the cleavage of Trop-2, wild-type Trop-2, and phosphorylation-blocked Trop-2 tagged with FLAG at the N-terminal end were further modified by adding a HA tag at the C-terminal end. FLAG-and HA-tagged wild-type Trop-2 and phosphorylation-blocked Trop-2 were immunoprecipitated from lysates of FLAG- and HA-tagged wild-type Trop-2 (WT-HA) and phosphorylation-blocked Trop-2 (SA-HA) expressing HCT116 cells, respectively, and subjected to SDS-PAGE, followed by immunoblotting and detection with anti-HA- or FLAG-tag antibodies. A broad band with a molecular mass of 50∼75 kDa was detected with both antibodies in both WT-HA and SA-HA cells (Fig. 3*A*, lanes a, b, c, d). Other bands with molecular masses of ∼40 kDa, ∼20 kDa, and ∼13 kDa were detected with anti-HA antibodies but not with anti-FLAG antibodies in WT-HA cells (Fig. 3*A*, lanes a, b), whereas these bands were hardly detected in SA-HA cells with either anti-FLAG-tag antibodies or anti-HA-tag antibodies (Fig. 3*A*, lanes c, d). These results suggest that the smaller three bands in WT-HA cells (Fig. 3*A*, lane b) were C-terminal peptides produced through cleavage of Trop-2 after its phosphorylation. In relation to Trop-2 phosphorylation, lysates of WT-HA cells were also analyzed by immunoblotting using anti-phospho-Trop-2 antibodies. Trop-2 C-terminal fragments corresponding to two bands with molecular masses of ∼40 kDa and ∼13 kDa were detected, but the ∼20-kDa band was not found probably due to its undetectable level (Fig. 3*A*, lane e). This result suggests that the phosphorylation of Trop-2 occurred before but not after the cleavage of Trop-2, this being consistent with the result that phosphorylation of Trop-2 may play an important role in the cleavage of Trop-2. In this context, furthermore, we investigated the relationship of Trop-2 phosphorylation with its cleavage. PKC-dependent phosphorylation of Trop-2 was demonstrated in the treatment of WT cells with PMA and PKC inhibitor Gö6983 as described previously (23). After treatment with PMA or 4α-PMA (control) for several hours, cell lysates were subjected to SDS-PAGE, followed by immunoblotting. Cleavage of Trop-2 was chased by the detection of a ∼13 kDa fragment with anti-HA antibodies. Cleavage of Trop-2 was enhanced by PMA treatment, peaking at 6 h (Fig. 3*B*). After treatment with Gö6983 or DMSO (control), the WT-HA cells were cultured in the presence of PMA or 4α-PMA for an additional 6 h, followed by detection of the Trop-2 fragment as described above. Cleavage of Trop-2 was clearly reduced on the treatment with PKC inhibitor Gö6983, indicating that Trop-2 phosphorylation initiated or enhanced its cleavage (Fig. 3*C*). It has been reported that the intracellular fragment of Trop-2 associated with β-catenin plays a role in a signaling event, leading to the promotion of self-renewal and initiation of prostatic intraepithelial neoplasia (30). To investigate whether or not phosphorylation of Trop-2 affects its association with β-catenin, Trop-2 was immunoprecipitated from lysates of wild-type and mutated Trop-2 expressing HCT116 cells, and then subjected to SDS-PAGE, followed by immunoblotting and detection with anti-β-catenin antibodies. Similar levels of β-catenin were coimmunoprecipitated with Trop-2 in these cells, suggesting that the interaction of Trop-2 with β-catenin is not regulated through its phosphorylation (Fig. S1). Next, the distributions of the HA-tagged Trop-2 fragment and β-catenin in WT-HA cells were observed immunochemically after treatment with PMA or 4α-PMA. Both the HA-tagged Trop-2 fragment and β-catenin were accumulated in nuclei on the treatment with PMA (Fig. 3*D*). In the merged image, β-catenin colocalized with the HA-tagged Trop-2 fragment in the nuclei can be observed as white dots in Figure 3*D*. White dot–positive cells in the nuclei were counted, and the percentage of positive cells in total cells is shown as a histogram (Fig. 3*E*). PMA treatment increased the β-catenin colocalized with the HA-tagged Trop-2 fragment in nuclei. These results indicate that nuclear transport of the Trop-2 fragment associated with β-catenin was enhanced by increased cleavage due to Trop-2 phosphorylation.

Furthermore, the accumulation of nuclear β-catenin, which is a final down-stream event of Trop-2 mediated signaling, may be critical to indicate the significance of Trop-2–mediated signaling. Recently, Zhao *et al.* (31) also reported that Trop-2 increased the accumulation of β-catenin in the nucleus to accelerate metastasis in gastric cancer. Thus, we tried to compare the levels of nuclear β-catenin by staining with anti-β-catenin antibodies among four cell types (Fig. 3, *F* and *G*). It was noted that the levels of nuclear β-catenin in Mock cells and SA cells were negligible, low in WT cells, and markedly high in SE cells (Fig. 3, *F* and *G*). Negligible levels of nuclear β-catenin in Mock cells and phosphorylation-blocked SA cells are consistent with the report that HCT116 cells have low levels of nuclear β-catenin (32). These results suggest that Trop-2–mediated signaling may occur weakly in WT cells and prominently in phosphomimetic SE cells and be blocked in SA cells. In fact, PMA treatment of WT cells increased ZEB1 expression and decreased E-cadherin expression. In contrast, Twist1 was hardly enhanced on the treatment with PMA (Fig. S2).

### β-Catenin–dependent expression of ZEB1

**Figure 4:**
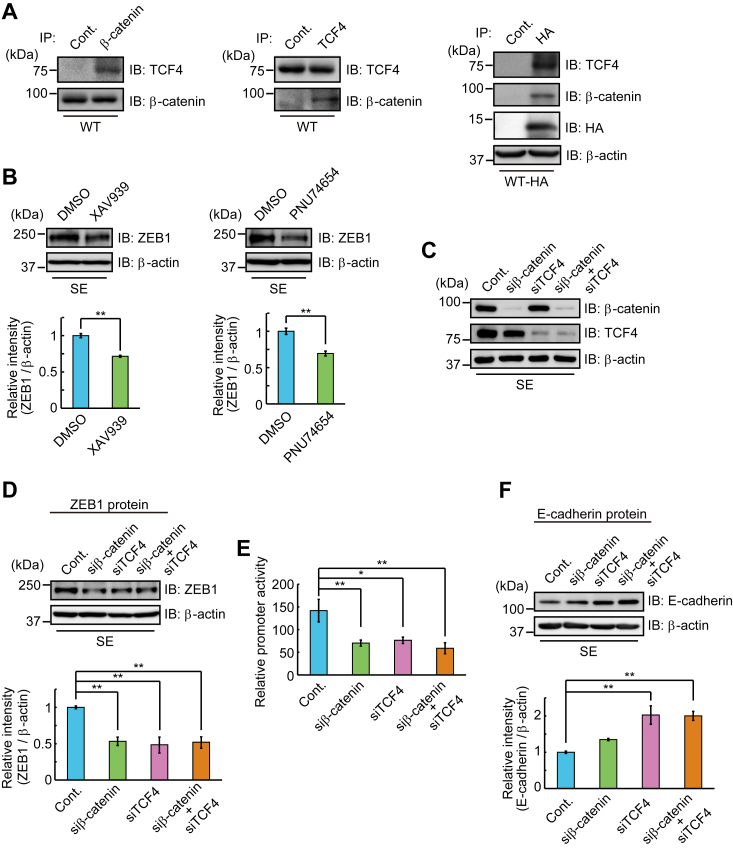
**β-Catenin-dependent expression of ZEB1.***A*, β-catenin (*left*) or TCF4 (*center*) was immunoprecipitated from lysate of WT cells, and coimmunoprecipitated TCF4 or β-catenin was detected by SDS-PAGE and immunoblotting. (*right*) Immunoprecipitates from lysates of WT-HA cells with anti-HA-tag antibodies were subjected to SDS-PAGE, followed by immunoblotting and detection of β-catenin and TCF4. *B*, lysates of SE cells treated with β-catenin inhibitors or DMSO (control) as described under Experimental Procedures were subjected to SDS-PAGE, followed by immunoblotting, and detection of ZEB1. β-Actin served as a loading control. The densities of bands were determined, and the ratio of ZEB1 to β-actin was calculated. That in SE cells treated with DMSO was taken as 1 (means ± S.E., n = 3, ∗∗*p* < 0.01). *C*, SE cells were treated with β-catenin and/or TCF4 siRNAs or control siRNA for 24 h, and the cell lysates were subjected to SDS-PAGE, followed by immunoblotting and detection of β-catenin and TCF4. β-Actin served as a loading control. *D*, lysates of SE cells treated with β-catenin and/or TCF4 siRNAs as described above were subjected to SDS-PAGE, followed by immunoblotting, and detection of ZEB1, and the densities of bands were determined. The ratio of ZEB1 to β-actin was calculated and that in SE cells treated with control siRNA was taken as 1. β-Actin served as a loading control (means ± S*.*E*.*, n = 3, ∗∗*p* < 0.01). *E*, SE cells were treated with β-catenin and/or TCF4 siRNAs as described above, and ZEB1 promoter activity was determined by using luciferase assay as described under Experimental Procedures. The value for SE cells treated with control siRNA was taken as 1 (means ± S.E., n = 3, ∗*p* < 0.05, ∗∗*p* < 0.01). *F*, lysates of SE cells treated with β-catenin and/or TCF4 siRNAs as described above were subjected to SDS-PAGE, followed by immunoblotting and detection of E-cadherin. The densities of bands were determined, and the ratio of E-cadherin to β-actin was calculated. The value in SE cells treated with control siRNA was taken as 1. β-Actin served as a loading control (means ± S.E., n = 3, ∗∗*p* < 0.01).

It has been reported that nuclear β-catenin binds to members of the TCF/LEF transcription factor family, and β-catenin/TCF4 binds to the human ZEB1 promoter, triggering its transcription (32). The formation of the β-catenin/TCF4 complex was confirmed by their coimmunoprecipitation in WT cells (Fig. 4*A*, *left* and *center*). Association with the Trop-2 fragment with β-catenin and TCF4 was also confirmed by coimmunoprecipitation with anti-HA-tag antibodies in WT-HA cells (Fig. 4*A*, *right*). ZEB1 was highly expressed in SE cells, but not in other Trop-2–expressing cells (Fig. 2, *A* and *B*). Thus, SE cells were treated with a β-catenin inhibitor (XAV939, PNU74654) for 24 h, and then the level of ZEB1 was determined by SDS-PAGE and immunoblotting of cell lysates. About 30% of ZEB1 protein decreased on the treatment with the β-catenin inhibitors (Fig. 4*B*). The decreases of the β-catenin and TCF4 proteins on the treatment with each siRNA were confirmed (Fig. 4*C*). Knockdown of β-catenin and/or TCF4 caused about a 50% decrease in the ZEB1 protein level (Fig. 4*D*). An equivalent decrease of ZEB1 promoter activity was confirmed by luciferase assay (Fig. 4*E*). It was also noted that the expression of E-cadherin increased on the knockdown of these factors (Fig. 4*F*). The reason why the level of E-cadherin protein was not elevated significantly in the treatment of SE cells with β-catenin siRNA is discussed later.

### STAT3-dependent expression of Twist1

**Figure 5:**
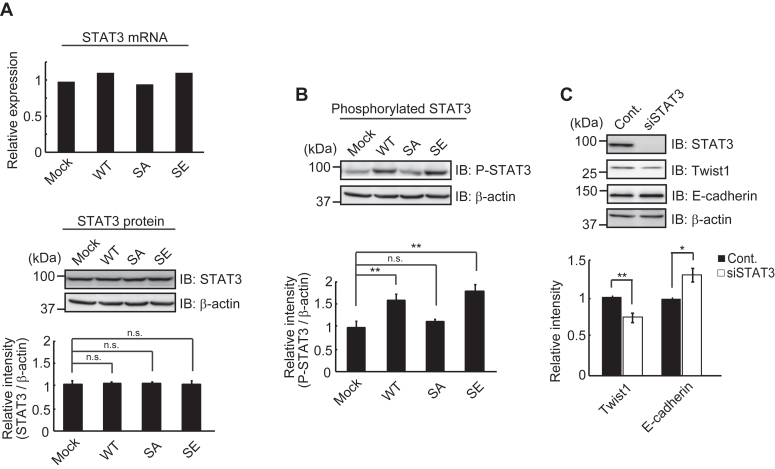
**Phosphorylated STAT3-dependent expression of Twist1.***A*, the levels of STAT3 mRNA in four cell types were determined by DNA microarray analysis (n = 1) and that in Mock cells was taken as 1. The levels of STAT3 protein in the four cell types were determined as described in Figure 1*D*, and the ratio of STAT3 to β-actin was calculated. The value in Mock cells was taken as 1. β-Actin served as a loading control (means ± S.E., n = 3, n.s.: not significant). *B*, phosphorylated STAT3 protein was detected with anti-phosphorylated STAT3 antibodies using the same blotted membrane as described in (*A*) after stripping of anti-STAT3 antibodies. Because STAT3, phosphorylated STAT3 and β-actin proteins were detected using the same blotted membrane, the β-actin image is shown as a loading control of both STAT3 (*A*) and phosphorylated STAT3 (*B*) proteins. The ratio of phosphorylated STAT3 to β-actin was calculated, and the value in Mock cells was taken as 1 (means ± S.E., n = 3, ∗∗*p* < 0.01, n.s.: not significant). *C*, WT cells were treated with STAT3 siRNA or control siRNA as described under Experimental Procedures. The levels of Twist1 and E-cadherin were determined as described in Figure 1*D*. The ratio of Twist1 and E-cadherin to β-actin was calculated, and the value in WT cells treated with control siRNA was taken as 1. β-Actin served as a loading control (means ± S.E., n = 3, ∗*p* < 0.05, ∗∗*p* < 0.01).

We also found that the levels of Twist1 mRNA in WT and SE cells were considerably higher than those in Mock and SA cells (Fig. 2, *A* and *B*). This finding suggests that Twist1 may be induced by downstream events through Trop-2 phosphorylation. Twist1 is transcriptionally induced by activation of STAT3 and mediates the STAT3 oncogenic function (33). In this context, we analyzed the levels of STAT3 protein and mRNA in wild-type and mutated Trop-2–expressing HCT116 cells. Although the levels of the STAT3 protein and mRNA were similar in these cells, phosphorylated (705Tyr) STAT3 was significantly elevated in WT and SE cells compared with Mock and SA cells (Fig. 5, *A* and *B*). It was also revealed that the phosphorylated STAT3 protein is dimerized and translocated to up-regulate the expression of Twist through its binding to the second proximal STAT3 binding site on the Twist promoter (33). In fact, the knockdown of STAT3 decreased the expression of Twist1 and reciprocally enhanced the expression of E-cadherin (Fig. 5*C*). Because similar levels of STAT3 were phosphorylated despite different signaling intensities between WT and SE cells, this STAT3-dependent pathway may be regulated by a STAT3 tyrosine kinase.

### Binding of galectin-3 to Trop-2 and subsequent signaling

**Figure 6:**
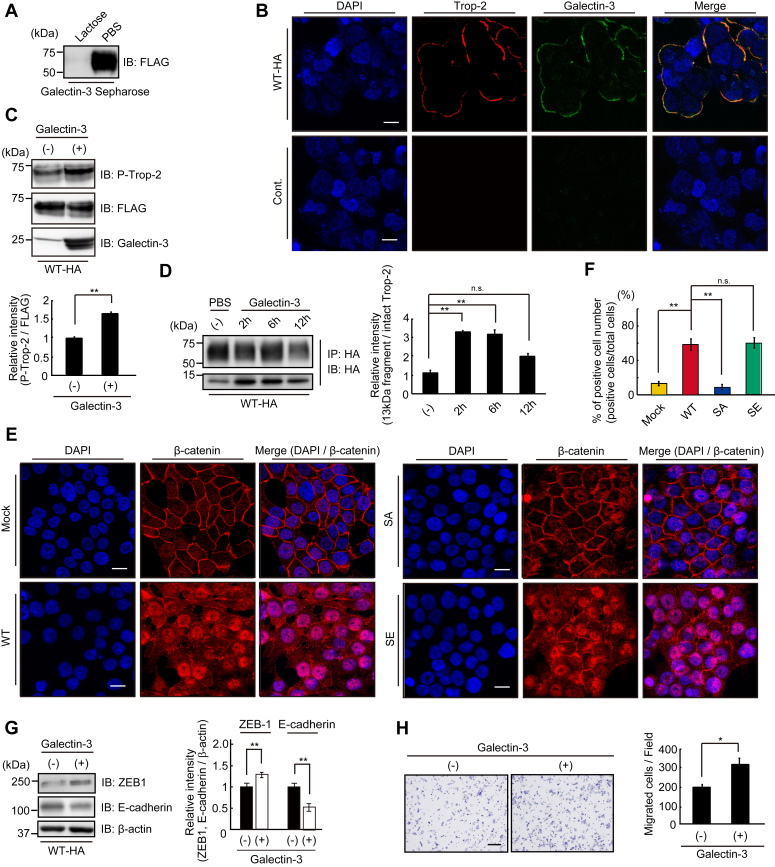
**Trop-2 mediated signaling triggered by binding of galectin-3 to Trop-2.***A*, lysates of WT-HA cells were incubated with galectin-3-Sepharose in the presence or absence of 50 mM lactose at 4 °C for 3 h. Galectin-3 binding proteins were subjected to SDS-PAGE, followed by immunoblotting and detection of FLAG-tagged Trop-2. *B*, galectin-3 (*green*) and FLAG-tagged Trop-2 (*red*) in WT-HA cells were immunostained as described under Experimental Procedures. Scale bars, 10 μm. *C*, WT-HA cells were treated with or without galectin-3 (40 μg/ml) for 30 min, and the cell lysates were subjected to SDS-PAGE, followed by immunoblotting, and detection of phosphorylated Trop-2 and FLAG-tagged Trop-2. The densities of bands were determined, and the ratio of phosphorylated Trop-2 to FLAG-tagged Trop-2 was calculated. The value in control cells was taken as 1 (means ± S.E., n = 3, ∗∗*p* < 0.01). *D*, WT-HA cells were treated with galectin-3 (40 μg/ml) for the indicated times. HA-tagged Trop-2 and its fragment were immunoprecipitated from the cell lysates and subjected to SDS-PAGE, followed by immunoblotting and detection of the intact Trop-2 and ∼13-kDa fragment. The ratio of HA-tagged ∼13 kDa fragment to HA-tagged intact Trop-2 was calculated, and the value in control experiment (PBS) was taken as 1 (means ± S.E., n = 3, ∗∗*p* < 0.01, n.s.: not significant). *E*, β-catenin in four cell types treated with galectin-3 (80 μg/ml) for 24 h was immunostained with anti-β-catenin antibodies (*red*) as described in Figure 3*F*. Nuclei were stained with DAPI (*blue*), and a representative photograph is shown. Scale bars, 10 μm. *F*, the ratio (%) of nuclear β-catenin positive cells per total cells in five randomly selected fields was calculated and is shown as a histogram (means ± S.E., n = 3, ∗∗*p* < 0.01, n.s.: not significant). *G*, WT-HA cells were cultured in the presence or absence of galectin-3 (40 μg/ml) for 24 h, and the levels of ZEB1 and E-cadherin protein were determined as described in Figure 1*D*. The ratio of ZEB1 and E-cadherin to β-actin was calculated. The value in control cells was taken as 1. β-Actin served as a loading control (means ± S.E., n = 3, ∗∗*p* < 0.01). *H*, migration of WT-HA cells treated with or without galectin-3 (80 μg/ml) as described above was evaluated by means of Transwell assays. A representative photograph of migrated cells is shown. The numbers of migrated cells per field were determined and is shown as a histogram. Scale bar, 100 μm (means ± S.E., n = 3, ∗*p* < 0.05).

A small part of Trop-2 was phosphorylated in WT cells, as reported in our previous paper (23). Cleavage of Trop-2 and transport of β-catenin to nuclei were detected in WT cells as described above. We speculated that ligation of an endogenous ligand with Trop-2 initiates this signaling and that there are two ways to elevate the phosphorylation of Trop-2. One is the growth-factor receptor-mediated phosphorylation of Trop-2. Expression of various growth factor receptors in HCT116 cells was revealed by DNA microarray analysis (Fig. S3*A*). Ligation of growth factors to their receptors is expected to activate receptor tyrosine kinase, resulting in activation of PKC. We examined the phosphorylation and subsequent cleavage of Trop-2 when WT cells were treated with various growth factors including EGF, FGF, TGF-β, HGF, and IGF-1. However, no growth factors exhibited a detectable effect on Trop-2 cleavage (Fig. S3*B*). Another possibility is that Trop-2 is phosphorylated directly on the binding of unknown extracellular ligands. Although we searched for a specific endogenous protein that binds to the ectodomain of Trop-2, no specific ligand was detected, as discussed later. Next, we examined the binding of galectin-3 to Trop-2, because Trop-2 is a highly glycosylated membrane protein, and Trop-2 and galectin-3 exhibited similar distribution patterns in various human cancer tissues (34). Lysates of WT cells were incubated with galectin-3-Sepharose in the presence or absence of 50 mM lactose. Galectin-3 binding proteins were examined on SDS-PAGE and immunoblotting. FLAG-tagged Trop-2 was detected in the galectin-3 binding proteins and its binding was completely abolished by lactose, indicating specific binding of galectin-3 to Trop-2 (Fig. 6*A*). Colocalization of Trop-2 and galectin-3 on the cell surface was observed immunochemically, suggesting the binding of galectin-3 to Trop-2 on the cell surface (Fig. 6*B*). After incubation of WT cells with galectin-3 for 30 min, FLAG-tagged Trop-2 was immunoprecipitated from the cell lysates, and then subjected to SDS-PAGE, followed by immunoblotting and detection of phosphorylated Trop-2 with anti-phosphorylated Trop-2 antibodies. The binding of galectin-3 to Trop-2 elevated the phosphorylation of Trop-2 (Fig. 6*C*). Subsequent cleavage of Trop-2 was also enhanced at 2∼6 h after treatment of the cells with galectin-3 (Fig. 6*D*). Next, we investigated whether or not the accumulation of nuclear β-catenin in four cell types increases through the binding of exogenously added galectin-3 to Trop-2 (Fig. 6, *E* and *F*). Expectedly, nuclear β-catenin in WT cells increased on the treatment with galectin-3 compared with that in WT cells without galectin-3 treatment, as shown in Figure 3*F*. In addition, the levels of nuclear β-catenin in SA cells and SE cells hardly changed regardless of the galectin-3 treatment. Those in SA cells were still negligible, probably due to the blocking of Trop-2–mediated signaling in Trop-2 phosphorylation-blocked cells. High levels of nuclear β-catenin in SE cells were maintained, suggesting that the maximum level of signaling occurs at all times in Trop-2 phosphomimetic cells.

We also investigated the effect of galectin-3 on the expression of ZEB1 and E-cadherin. Expectedly, expression of ZEB1 increased and, reciprocally, that of E-cadherin clearly decreased (Fig. 6*G*) at 24 h after incubation of the cells with galectin-3. Eventually, galectin-3–treated cells exhibited higher cell mobility compared with control cells (Fig. 6*H*).

In this context, we performed the same experiment using SA cells. Exogenously added galectin-3 had no effect on the expression of ZEB1 and E-cadherin (Fig. S4), consistent with the result that β-catenin was hardly accumulated in nuclei in SA cells (Fig. 6*E*). Actually, exogenously added galectin-3 enhanced ZEB1 transcription and subsequent down-modulation of E-cadherin in WT cells. It would be interesting to see whether or not endogenous galectin-3 could reduce E-cadherin expression. We observed four cell types cultured for 7 days microscopically (Fig. S5*A*). WT cells and SE cells appeared to be in contact more loosely compared with SA cells. Next, the expression of E-cadherin and cell surface galectin-3 was examined and compared using WT cells cultured for 1 and 7 days. Consistently, the level of E-cadherin in WT cells cultured for 7 days decreased compared with that in WT cells cultured for 1 day (Fig. S5*B*). WT cells cultured for seven cells were more densely stained with anti-galectin-3 antibodies compared with WT cells cultured for 1 day (Fig. S5*C*), suggesting that Trop-2–mediated signaling was elevated through accumulated cell surface galectin-3 in WT cells cultured for 7 days.

### Change in E-cadherin expression in MCF-7 cells and DU145 cells treated with PMA, galectin-3, or Trop-2 siRNA

**Figure 7:**
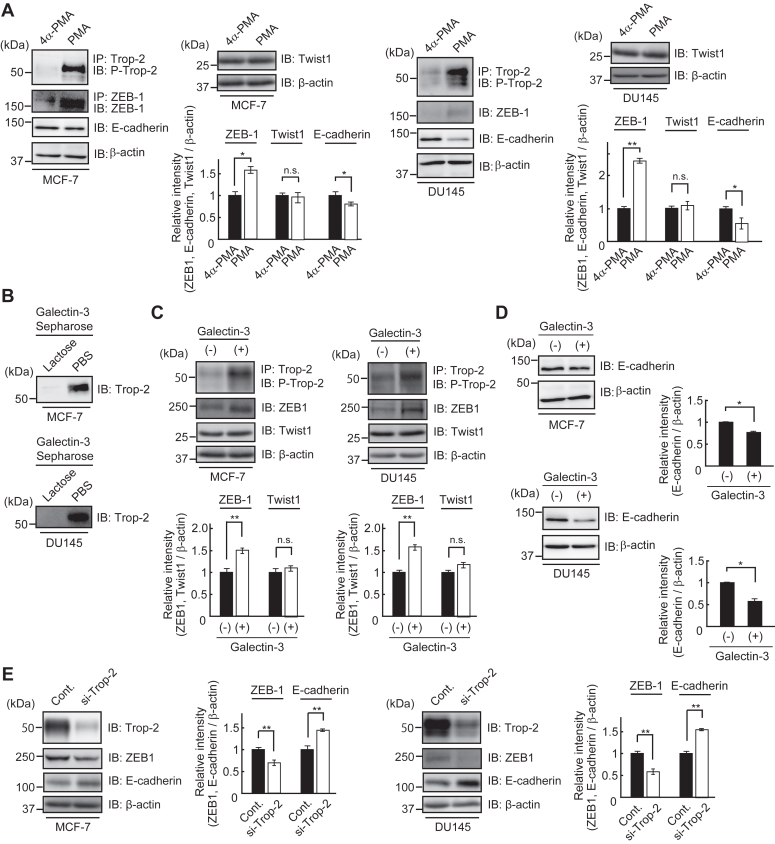
**Change of E-cadherin expression in MCF-7 cells and DU145 cells treated with PMA, galectin-3, or Trop-2 siRNA.***A*, the levels of phosphorylated Trop-2, ZEB1, Twist1, and E-cadherin were determined as described in Figure 1*D*. MCF-7 cells and DU145 cells were treated with 4α-PMA or PMA (100 ng/ml), and after 24 h, lysates were prepared to determine the levels of phosphorylated Trop-2, ZEB1, and Twist1. Similarly, the levels of E-cadherin were determined using lysates prepared after 48 h. β-Actin served as a loading control. The densities of bands were determined, and the ratios of ZEB1, Twist1, and E-cadherin to β-actin were calculated. The values in MCF-7 cells and DU145 cells treated with 4α-PMA were taken as 1 (means ± S.E., n = 3, ∗*p* < 0.05, ∗∗*p* < 0.01, n.s.: not significant). *B*, lysates of MCF-7 cells and DU145 cells were incubated with galectin-3-Sepharose in the presence or absence of 50 mM lactose, and galactose binding proteins were subjected to SDS-PAGE, followed by immunoblotting and detection with anti-Trop-2 antibodies. *C*, lysates of MCF-7 cells and DU145 cells treated with or without galectin-3 (40 μg/ml) for 24 h were subjected to SDS-PAGE and immunoblotting, followed by detection of phosphorylated Trop-2, ZEB1, Twist1, and β-actin. β-Actin served as loading control. The densities of bands were determined, and the ratios of ZEB1 and Twist1 to β-actin were calculated. The values in galectin-3 non-treated cells were taken as 1 (means ± S.E., n = 3, ∗∗*p* < 0.01, n.s.: not significant). *D*, lysates of MCF-7 cells and DU145 cells treated with or without galectin-3 (40 μg/ml) for 24 h were subjected to SDS-PAGE and immunoblotting, followed by detection with anti-E-cadherin and anti-β-actin antibodies. β-Actin served as a loading control. The densities of bands were determined, and the ratio of E-cadherin to β-actin was calculated. The values in galectin-3 non-treated cells were taken as 1 (means ± S.E., n = 3, ∗*p* < 0.05). *E*, MCF-7 cells and DU145 cells were treated with Trop-2 siRNA or control siRNA for 24 h, and cultured in the presence of galectin-3 (40 μg/ml) for 24 h. The cell lysates were subjected to SDS-PAGE, followed by immunoblotting and detection of Trop-2, ZEB1, E-cadherin, and β-actin. β-Actin served as a loading control. The densities of bands were determined, and the ratios of ZEB1 and E-cadherin to β-actin were calculated. The values in each cell treated with control siRNA was taken as 1 (means ± S.E., n = 3, ∗∗*p* < 0.01).

Trop-2 was immunoprecipitated from lysates of MCF-7 cells and DU145 cells and then subjected to SDS-PAGE and immunoblotting, followed by detection with anti-Trop-2 antibodies. A band with a molecular mass of about 50 kDa was detected (Fig. S6*A*). Expression of Trop-2 in MCF-7 cells and DU145 cells was also demonstrated by flow cytometry (Fig. S6*B*). Next, lysates were prepared from MCF-7 cells and DU145 cells treated with PMA or 4α-PMA and then subjected to SDS-PAGE and immunoblotting. Phosphorylated Trop-2, ZEB1, Twist1, E-cadherin, and β-actin were detected. Although the expression of Twist1 hardly changed, the level of ZEB1 was enhanced, and, reciprocally, the expression of E-cadherin was down-modulated on the treatment with PMA (Fig. 7*A*).

The binding of galectin-3 to Trop-2 expressed in MCF-7 cells and DU145 cells was investigated by using galectin-3-Sepharose as described in Figure 6*A*. Trop-2 was detected by SDS-PAGE and immunoblotting in the galectin-3 binding proteins in both cells (Fig. 7*B*). Trop-2 phosphorylation and expression of ZEB1 and Twist1 in MCF-7 cells and DU145 cells were also investigated by immunoblotting after treatment with exogenously added galectin-3. ZEB1 but not Twist1 increased in association with the enhancement of Trop-2 phosphorylation (Fig. 7*C*). Expectedly, the expression of E-cadherin was clearly down-modulated after treatment with galectin-3 (Fig. 7*D*).

Furthermore, in order to confirm the biological significance of Trop-2, we tried to knockdown Trop-2 in MCF-7 cells and DU145 cells. Decreased Trop-2 expression in MCF-7 cells and DU145 cells was confirmed by SDS-PAGE and immunoblotting. MCF-7 cells and DU145 cells treated with Trop-2 siRNA or control siRNA were cultured in the presence of galectin-3 for 24 h, and then cell lysates were subjected to SDS-PAGE and immunoblotting, followed by detection of Trop-2, ZEB 1, E-cadherin and β-actin. Downregulation of ZEB1 and reciprocal upregulation of E-cadherin were also demonstrated associated with Trop-2 siRNA treatment of MCF-7 cells and DU145 cells (Fig. 7*E*).

### Detection of FLAG-tagged Trop-2 in the liver and lung of nude mice bearing intraperitoneally or subcutaneously injected three cell types

**Figure 8:**
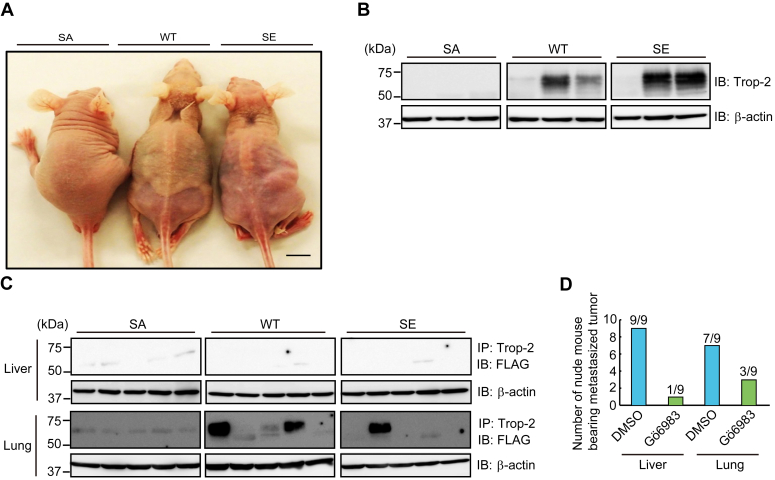
**Detection of FLAG-tagged Trop-2 in liver and lung of nude mice bearing intraperitoneally or subcutaneously injected three cell types.***A*, SA, WT, and SE cells (5 × 10^6^ cells) were injected intraperitoneally into nude mice, and after 1 month, the appearance of tumor-bearing mice was observed. Scale bar, 10 mm. *B*, livers were harvested from the tumor-bearing mice. Trop-2 was immunoprecipitated from the liver extracts, which were subjected to SDS-PAGE, followed by immunoblotting and detection of FLAG-tagged Trop-2. With respect to WT and SE cells, typical samples of FLAG-tagged Trop-2-detected or non-detected nude mice are shown. The occurrence of FLAG-tagged Trop-2-detected liver was 2/5, 2/4, and 0/3 of WT, SE, and SA cell-inoculated mice, respectively. *C*, three cell types, as described in Figure 8*A*, were injected subcutaneously into the backs of nude mice. After 2 months, livers and lungs were harvested, and detection of FLAG-tagged Trop-2 in the livers and lungs was performed as described above. Although FLAG-tagged Trop-2 was not detected in liver, FLAG-tagged Trop-2 in lung was detected in 3/5, 1/5, and 0/5 of WT, SE, and SA cell-inoculated mice, respectively. *D*, WT cells (5 × 10^6^ cells) were injected intraperitoneally into nude mice. Starting 1 day after tumor cell injection, nude mice received daily intraperitoneal injections of Gö6983 (0.25 mg/mouse) or DMSO. After 4 weeks, the mice were euthanized, and their livers and lungs were harvested. Thereafter, FLAG-tagged Trop-2 was detected in the extracts of livers and lungs. Histograms show the numbers of FLAG-tagged Trop-2-detected nude mice.

Next, to examine the cell behavior of the three cell types *in vivo*, these cells were injected into nude mice intraperitoneally or subcutaneously. Intraperitoneally injected cells gave rise to bloody ascites regardless of being wild-type or mutated Trop-2 expressing cells, however, the appearance of nude mice was extremely different between SA and the other two cells (Fig. 8*A*). Livers and lungs were harvested at 4 weeks after tumor inoculation, and immunoprecipitation of FLAG-tagged Trop-2 from tissue extracts and its detection were performed as described under Experimental Procedures section. FLAG-tagged Trop-2 was detected in two livers and four lungs of five WT cell-inoculated nude mice, and in two livers and three lungs of four SE cell-inoculated nude mice, whereas FLAG-tagged Trop-2 was not detected in either liver or lung of nude mice inoculated with SA cells (Fig. 8*B*).

Subcutaneously injected cells formed tumors apparently at similar rates among these cells. At 8 weeks after subcutaneous injection, the nude mice were euthanized, and then their primary tumors, livers, and lungs were harvested. FLAG-tagged Trop-2 was detected in some lungs but not in livers (Fig. 8*C*). FLAG-tagged Trop-2 was detected in three lungs and one lung of five nude mice inoculated with WT cells and SE cells, respectively. However, even in the lungs or livers in which FLAG-tagged Trop-2 was detected, metastatic colonies were not observed histologically, probably due to the undetectable size of tumor cell clusters. In contrast, no FLAG-tagged Trop-2 was detected in the lungs of nude mice inoculated with SA cells (n = 5). In any case, SA cells seem to be unable to move to secondary sites, suggesting that phosphorylation of Trop-2 may be linked to the acquisition or enhancement of metastatic potential.

Therefore, we examined the effect of PKC inhibitor Gö6983 on the mobility of WT cells. WT cells were injected intraperitoneally into nude mice. Starting 1 day after tumor cell injection, nude mice received daily intraperitoneal injection of Gö6983 (0.25 mg/mouse). After 4 weeks, FLAG-tagged Trop-2 was detected in the liver and lung tissues as described above. FLAG-tagged Trop-2 in the liver was detected in nine of nine control mice and two of nine of Gö6983-treated mice. FLAG-tagged Trop-2 in lung was detectable in seven of nine of control mice and three of nine of Gö6983-treated mice, indicating that Gö6983 inhibited the mobility of WT cells effectively (Fig. 8*D*).

### Distributions of E-cadherin, phosphorylated Trop-2, and Trop-2 in gastric cancer tissues

**Figure 9:**
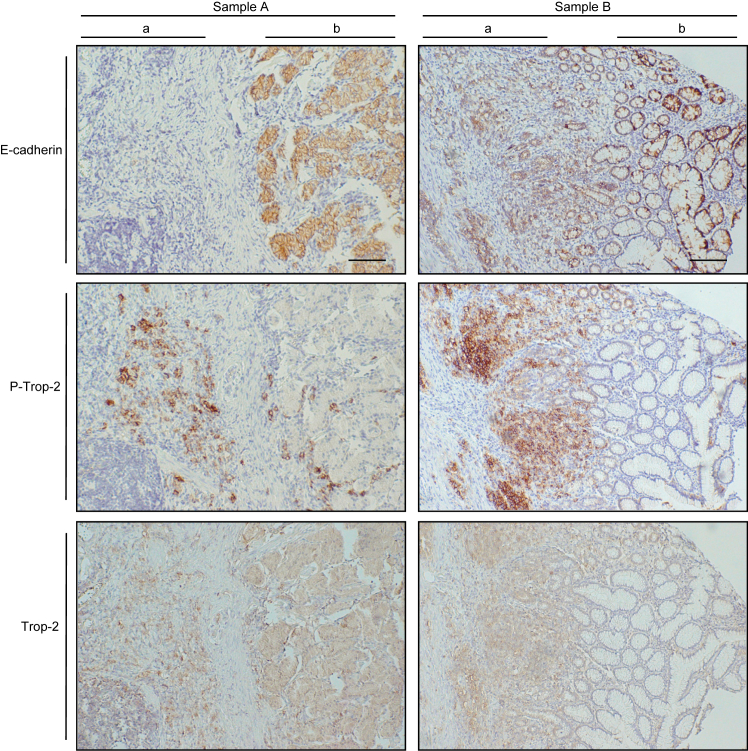
**Distributions of E-cadherin and phosphorylated Trop-2 in gastric cancer tissues.** Gastric cancer tissues (20 cases) were immunostained with anti-E-cadherin, anti-phosphorylated Trop-2, and anti-Trop-2 antibodies and observed immunohistochemically. Nuclei were stained with hematoxylin. Typical specimens including histologically normal appearing and cancerous tissues are shown. Scale bars, 100 μm. (*A*) malignant lesion, and (*B*) adjacent non-malignant lesion.

Gastric cancer tissues (20 cases) were immunostained. Phosphorylated Trop-2, Trop-2, and E-cadherin–positive tissues were found in 8, 10, and 10 cases, respectively. Immunohistochemical staining of typical specimens including histologically normal appearing and cancerous tissues is demonstrated in Figure 9. E-cadherin was expressed mainly at the cell membrane. Intense E-cadherin staining was observed in normal-appearing tissues, whereas in cancerous tissues, the staining was reduced. On the other hand, phosphorylated Trop-2 was highly expressed in cancerous tissues, and adjacent normal-appearing tissues were only faintly stained. Similar expression levels of Trop-2 were observed in normal and cancerous tissues, suggesting a possibility that the phosphorylation of Trop-2 may be correlated with the down-modulation of E-cadherin. Further studies are necessary to clarify statistically the relation of E-cadherin expression with phosphorylated Trop-2 in cancer tissues.

## Discussion

Although it has been considered that growth factors such as TGF-β play a predominant role in the promotion of EMT (1), growth factors secreted on the apical side may be unable to ligate to basolaterally localized receptors until the deconstruction of cell-cell junctions. Consistently, TGF-β is produced mainly in stromal fibroblasts after the formation of the tumor tissue microenvironment (35). To better characterize the EMT cascade temporally and spatially, it is important to understand how intercellular contacts change during early EMT. First, we focused on the function of Trop-2 with respect to the maintenance of tight junctions, because Trop-2 and its closest homolog, EpCAM, are required for the stability and proper distribution of claudin-1 and claudin-7 in cornea and intestinal epithelial tissues, respectively (20, 21, 22). In a previous paper (23), we demonstrated that phosphorylation of Trop-2 leads to mislocalization and posttranslational reduction of claudin-7. In this study, we prepared HCT116 cells expressing wild-type and mutated Trop-2 with Ala and Glu instead of Ser322 to block and mimic the phosphorylation of Trop-2, respectively, and a migration assay was performed using Transwell. Trop-2 phosphorylation-blocked cells (SA cells) exhibited significantly less motility compared with wild-type and phosphomimetic Trop-2 expressing cells (WT, SE cells). It is well-known that down-modulation of E-cadherin transcription is a primary inducer of the onset of EMT. Expectedly, E-cadherin mRNA and protein were markedly reduced in Trop-2 phosphomimetic cells (SE cells) (Fig. 1), indicating that expression of E-cadherin is regulated transcriptionally through Trop-2 mediated signaling, consistent with the fact that ZEB1, which is one of the major proteins that repress E-cadherin expression transcriptionally, was markedly elevated in SE cells (Fig. 2). Expression of vimentin and N-cadherin, which are mesenchymal markers, was slightly increased in SE cells (Fig. S7), suggesting that SE cells may be an intermediate state of EMT.

With respect to Trop-2 mediated signaling, it is reported that the Trop-2 cytoplasmic fragment is translocated to nuclei with β-catenin, resulting in upregulation of downstream targets cyclin D1 and c-myc (30). Thus, cleavage of Trop-2 seems to be critical for signaling. Cleavage of Trop-2 was enhanced by the activation of PKCα/δ on the treatment with PMA and conversely down-modulated by treatment with Gö6983, suggesting that phosphorylation of Trop-2 may be essential for its cleavage or make it more susceptible to cleavage enzyme, probably due to its conformational change (Fig. 3). Consistently, it has been reported that novel PKC activity is required for TACE-dependent cleavage of Trop-2 (36). However, PMA and Gö6983 are not specific to PKCα/δ and low levels of C-terminal fragments were detected even in the presence of Gö6983 (Fig. 3*C*). Maetzel *et al.* (37) reported that EpCAM also functions in cellular transformation *via* RIP cleavage mechanisms. Although EpCAM is the closest homolog of Trop2 (17), it does not possess a phosphorylation site corresponding to Ser322 of Trop-2. Thus, although it is clear that phosphorylation of Trop-2 enhances its cleavage, there remains a possibility that Trop-2 may be cleaved through an other mechanism irrespective of phosphorylation.

A similar level of β-catenin was coimmunoprecipitated with Trop-2 in wild-type and mutated Trop-2 expressing cells, suggesting no effect of phosphorylation on the interaction of Trop-2 with β-catenin. The C-terminal portion of EpCAM forms a nuclear complex with β-catenin (37) as well as Trop-2, consistent with this result that β-catenin binds to Trop-2 regardless of the Trop-2 phosphorylation. It has been also noted that in normal epithelial cells, β-catenin may be sequestered through its binding to the cytoplasmic domain of Trop-2 like E-cadherin. Thus, the increased accumulation of β-catenin and Trop-2 fragments in nuclei on PMA treatment may be due to the enhanced cleavage of Trop-2. Accumulation of nuclear β-catenin in four cell types seems to be closely related to the phosphorylation of Trop-2. In relation to the accumulation of nuclear β-catenin, ZEB1 was induced in SE cells but not in WT cells (Fig. 1, *C* and *D*), probably consistent with the result that the level of nuclear β-catenin in WT cells was considerably lower than that in SE cells. Thus, we speculate that the signaling intensity in WT cells was too low to induce ZEB1 transcription.

β-Catenin is known to bind DNA *via* TCF4 and the β-catenin/TCF4 complex binds to the ZEB1 promoter (32), consistent with the results that similar levels of ZEB1 were decreased by knockdown of β-catenin, TCF4, or even both β-catenin and TCF4 (Fig. 4*D*). Furthermore, E-cadherin increased by knockdown of TCF4, but the knockdown of β-catenin did not enhance E-cadherin expression significantly (Fig. 4*F*). In this context, we examined the effect of β-catenin knockdown on the expression of E-cadherin mRNA in SE cells. Expectedly, the levels of E-cadherin mRNA in SE cells treated with β-catenin siRNA were elevated about 1.6 fold compared with those in SE cells treated with control siRNA, as shown in Fig. S8. These results suggest that the stability of E-cadherin may be down-modulated posttranslationally. It is well-known that the cytoplasmic C-terminal region of E-cadherin binds to β-catenin. The “PEST” sequence of E-cadherin is subjected to rapid turnover *via* the action of ubiquitin ligases. However, this motif overlaps with the β-catenin binding region, this preventing proteasomal degradation of E-cadherin when bound to β-catenin (38). Thus, we considered that knockdown of β-catenin may lead to decrease E-cadherin stability. In this experiment, ∼80% of β-catenin and ∼90% of TCF-4 were knockdowned. In addition, DNA microanalysis revealed that expression levels of β-catenin mRNA were higher than those of TCF4 mRNA in SE cells. Knockdown of β-catenin in SE cells could decrease E-cadherin associated with β-catenin. In addition, binding of β-catenin remaining in the cell to TCF4/Trop-2 C-terminal fragment complex could further decrease β-catenin-associated E-cadherin, resulting in prominent degradation of E-cadherin. On the other hand, in SE cells treated with β-catenin and TCF4 siRNAs, β-catenin remained in the cell and maybe could bind to E-cadherin due to the knockdown of TCF4. Further study is necessary to confirm this speculation.

Twist1 was elevated in WT and SE cells, but not in Mock and SA cells, suggesting that its induction is a downstream event through Trop-2 mediated signaling as well as the induction of ZEB1. Twist is known to be induced transcriptionally by STAT3 (39). Expectedly, the knockdown of STAT3 decreased the expression of Twist1, and activated STAT3, which is phosphorylated at Tyr705, was elevated in WT and SE cells, but not in Mock and SA cells (Fig. 5). STAT3 phosphorylation seems to be a critical event in this pathway. In contrast with different signaling intensities in WT and SE cells, as described above, the similar levels of phosphorylated STAT3 in both cell types (Fig. 5*B*) suggest that signaling intensity may be regulated by STAT3 tyrosine kinase. In addition, in contrast with the results that ZEB1 was increased and E-cadherin was decreased on the treatment with PMA (Fig. S2), further enhancement of Twist1 was not induced in WT cells on the treatment with PMA. Although this pathway seems to be non-functional substantially, there is a possibility that it works co-operatively with other signaling pathways *via* STAT3 phosphorylation. STAT3 is known to be phosphorylated by Janus kinases, growth factor receptor tyrosine kinases, and Src family tyrosine kinases (33). It is also reported that Src is associated with STAT3 in v-Src-transformed cell lines (40). These tyrosine kinases phosphorylate STAT3 through the engagement of cytokines and growth factors with each receptor. When Trop-2 mediated pathway works co-operatively with these receptors, it could enhance STAT3 phosphorylation additively.

Thus, Trop-2 phosphorylation may be a key step in the regulation of the expression of ZEB1. Therefore, it is interesting to determine by which mechanism PKCα/δ could be activated. One possibility is growth factor receptor-mediated activation of PKC. PKC is activated through down-stream events initiated by the ligation of growth factors to their receptors. We confirmed the expression of various receptors for EGF, FGF, TGF, PDGF, and IGF in HCT116 cells by DNA microarray analyses (Fig. S3). However, no growth factors had a detectable effect on the Trop-2 phosphorylation and cleavage, indicating that growth factor-triggered EMT is not involved in this EMT-promoting cascade.

Therefore, we searched for a ligand that binds to Trop-2 and leads to the phosphorylation of Trop-2. However, a specific endogenous ligand was not detected on affinity chromatography using Sepharose conjugated with a polypeptide of the Trop-2 ectodomain. Next, we examined the binding of Trop-2 to endogenous lectins, because Trop-2 is heavily glycosylated, and thereby galectin-3 was detected. The level of galectin-3 is correlated with tumor progression, and the concentration of circulating galectin-3 in the serum of cancer patients has been reported to be higher than that in healthy individuals (41), and a higher serum galectin-3 level was detected in patients with metastasis arising from prostate cancer (42). Galectin-3 is expressed in a variety of normal epithelial cells and secreted predominantly to the apical side. This lectin is recycled by the cells and plays a crucial role in apical protein trafficking (43, 44). Thus, a galectin-3-triggered cascade to induce EMT may be possible spatially from the initial stage before the deconstruction of cell polarity. In addition, because galectin-3 can act as a bivalent or multivalent ligand (45), it can cross-link cell surface glycoconjugates, which can trigger strong transmembrane glycoprotein-mediated signaling (46). The binding of galectin-3 to N-acetyllactosamine residues bearing on a variety of glycoconjugates gives rise to diverse biological effects. In fact, galectin-3 interacts with FGFR (47), TGF-R (48), Notch1 (49, 50), MUC1 (51), and integrins (52), and triggers a variety of signalings, which may be of great advantage for tumor progression. Interestingly, binding of galectin-3 to Notch1 also triggers the cleavage of Notch1 by the same enzymes as for Trop-2 cleavage, followed by a similar signaling pathway involving nuclear translocation of the Notch1 intracellular fragment.

**Figure 10:**
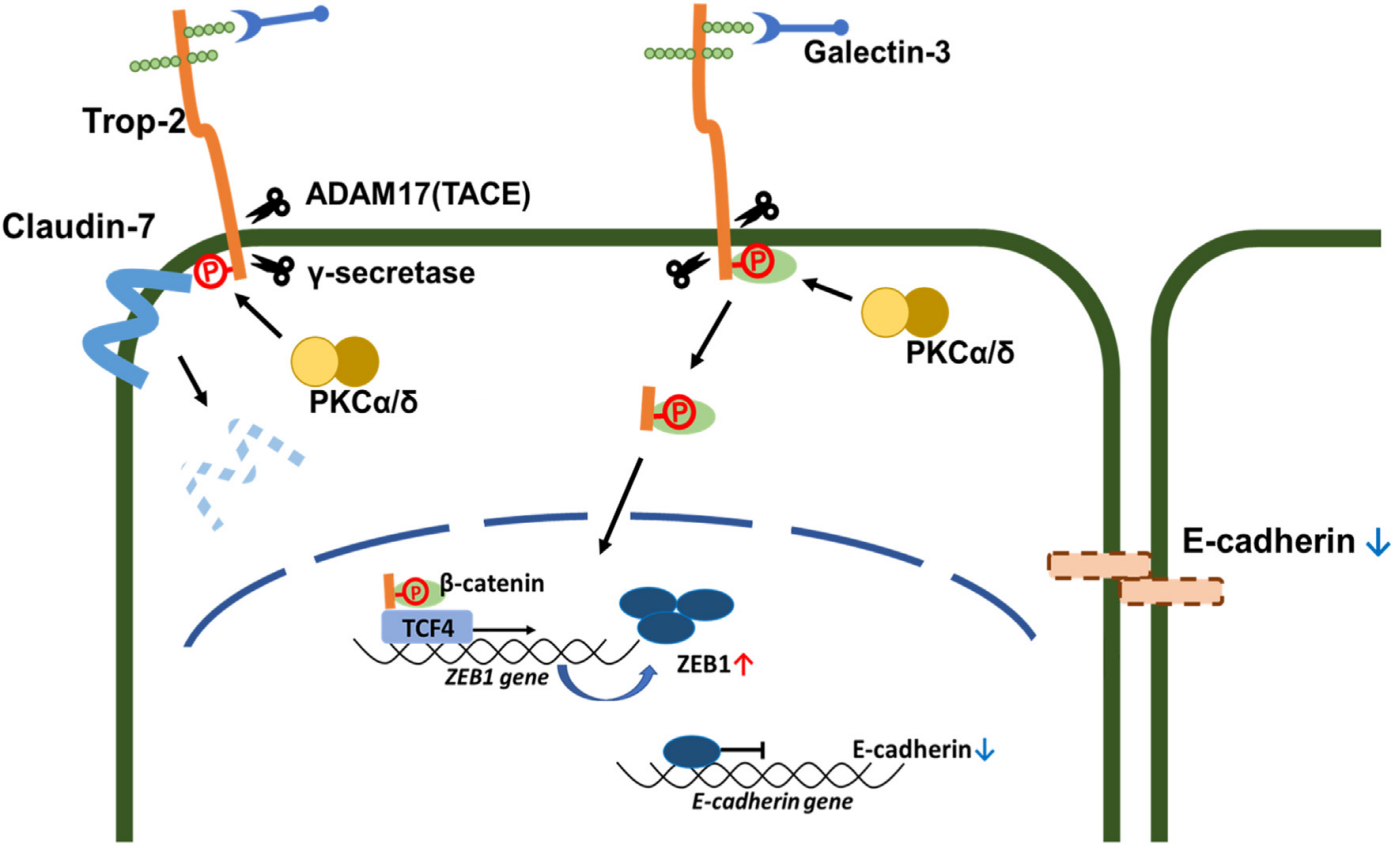
**Schematic model of Trop-2-driving derangement of claudin-7 and E-cadherin.** It is noted that both tight and adherence junctions are deconstructed simultaneously through Trop-2–mediated pathway.

Furthermore, Trop-2–mediated tumor progression was investigated by means of *in vivo* experiments. Wild-type and phosphomimetic Trop-2 but not phosphorylation-blocked Trop-2 were detected in the lung and/or liver of some nude mice bearing primary ascites or subcutaneous tumors, suggesting the relation of Trop-2 phosphorylation with cell mobility (Fig. 8). Metastasis is a complex process including dissemination of tumor cells from primary sites, transportation of tumor cells, and settlement and re-growth of tumor cells in secondary sites. Our present study indicates that down-modulation of E-cadherin expression through Trop-2-mediated signaling may play a critical role in early metastatic events at the primary site. However, although FLAG-tagged Trop-2 was detected biochemically at secondary sites, tumor cell clusters were not observed histologically. According to the American Joint Committee (AJCC), tumor cell clusters less than 2.0 mm are called micrometastasis, and clusters smaller than 0.2 mm and single cells are referred to as independent tumor cells (53). Thus, FLAG-tagged Trop-2 may be derived from independent tumor cells. At present, it is uncertain whether or not the settlement and regrowth of tumor cells at a secondary site are affected or regulated by Trop-2. Thus, although the significance of Trop-2 through all the steps of metastasis has not been elucidated, our findings indicate that derangement of epithelial cell attachment through Trop-2 phosphorylation may play an important role in tumor cell metastasis. A schematic model of Trop-2 mediated signaling cascade is shown in Figure 10. Binding of galectin-3 induced phosphorylation of Trop-2 and subsequent cleavage of Trop-2 occurred, which was linked to mislocalization of claudin-7 and a decrease of its stability probably due to loss of interaction between claudin-7 and Trop-2 as described previously. Furthermore, its C-terminal fragment was transported to nuclei, and its complex with β-catenin and TCF4 enhanced the transcription of ZEB1. The Trop-2–mediated pathway may play a critical role in driving EMT and subsequent metastasis because both tight and adherence junctions are deconstructed simultaneously, and this cascade is spatially functional from the premalignant stage.

## Experimental procedures

### Antibodies

The following antibodies were used: mouse anti- FLAG M2 (Sigma-Aldrich), rabbit anti-HA tag (Abcam), rabbit anti-E-cadherin (Cell Signaling Technology), mouse anti-E-cadherin (DAKO), rabbit anti-ZEB1 (Cell Signaling Technology), rabbit anti-Snail1 (Cell Signaling Technology), rabbit anti-Twist1 (BIORAD), mouse anti-β-catenin (Santa Cruz Biotechnology), mouse anti-TCF4 (Santa Cruz Biotechnology), mouse anti-β-catenin (Sigma-Aldrich), rabbit anti-phospho-STAT3 (Abcam), rabbit anti-STAT3 (Abcam), rabbit anti-galectin-3 (Abcam), goat anti-Trop-2 (R&D), and HRP-conjugated mouse anti-FLAG M2 antibodies (Sigma-Aldrich). Rabbit anti-phospho-Trop-2 antibodies were prepared as described previously (23).

### Cells

A human colorectal cancer cell line, HCT116 cells, a human breast cancer cell line, MCF-7 cells, and a human prostate cancer cell line, DU145 cells, were obtained from the American Type Culture Collection (Manassas). HCT116 cells, their Trop-2 transfectants, and MCF-7 cells were cultured in DMEM containing 10% heat-inactivated FBS, 4 mM L-glutamine, 100 units/ml penicillin, and 100 μg/ml streptomycin. DU145 cells were cultured in RPMI1640 containing 10% heat-inactivated FBS, 4 mM L-glutamine, 100 units/ml penicillin, and 100 μg/ml streptomycin. HCT116 cells expressing FLAG-tagged wild-type Trop-2 (WT cells) and Trop-2 mutated with Ala or Glu instead of Ser322 (SA or SE cells), and mock cells (Mock cells) were prepared and cultured as described previously (23). The four cell types (Mock, WT, SA, and SE cell) were prepared as follows unless otherwise described. Confluent cells were dispersed with trypsin treatment. After washing with a culture medium, cells were seeded and cultured for 1 day.

HCT116 cells expressing wild-type and phosphorylation-blocked Trop-2 with FLAG and HA tags at the N- and C-terminals (WT-HA cells, SA-HA cells), respectively, were prepared as follows: FLAG-tagged wild-type Trop-2 and phosphorylation-blocked Trop-2 plasmids, as described previously (23), were modified by introducing a HA tag at the C-terminus using a KOD-Plus-Mutagenesis Kit (TOYOBO), and the following primers: 5′-GTT CCA GAT TAC GCT TAG GTA CCC GGC GGG GCA GGG GAT G-3′ and 5′-ATC GTA TGG GTA CAA GCT CGG TTC CTT TCT CAA CTC C-3′. After sequencing and purification, the plasmids were transfected into HCT116 cells using FuGENE HD Transfection Reagent (Promega) according to the manufacturer’s instructions, and then selected with 600 μg/ml G418.

### DNA microarray analysis

The mRNA levels of several genes were determined by DNA microarray analysis as described previously (23).

### Flow cytometry

MCF-7 cells and DU145 cells were incubated with mouse anti-Trop-2 antibodies or the mouse isotype IgG (IgG2a). After incubation with fluorescein isocyanate (FITC)-conjugated secondary antibodies, the cells were analyzed by flow cytometry.

### Immunoprecipitation and immunoblotting

Cell extracts were prepared basically as described previously (23). In brief, subconfluent cells were lysed with a solubilizing solution (25 mM Tris-HCl, pH7.5, 150 mM NaCl, 5 mM EDTA, 1% Triton X-100 (TX-100)) containing protease and phosphatase inhibitor cocktails (Nakarai Tesque), and subsequently sonicated. After centrifugation at 20,000*g* at 4 °C for 10 min, the supernatants were used as cell extracts. To detect Trop-2 in lung and liver tissues harvested from tumor-bearing nude mice, lungs and livers were cut into small pieces, and after washing with PBS, tissue extracts were prepared as described above. Trop-2 in the cell extracts was immunoprecipitated using anti-FLAG M2 Magnetic Beads (Sigma-Aldrich) or anti-HA-tag mAb-Magnetic Agarose (Medical and Biological Laboratories). Trop-2 in the tissue extracts was immunoprecipitated using goat anti-Trop-2 antibodies and protein G-SepharoseTM4 Fast Flow (GE Healthcare). The immunoprecipitates and cell extracts were subjected to SDS-PAGE, followed by immunoblotting. The target proteins were detected as bands by chemiluminescence, and the densities of the bands were determined with Image J software (National Institutes of Health).

### Immunocytochemistry

Wild-type or mutated Trop-2 expressing HCT116 cells were stained immunocytochemically with anti-FLAG-tagged Trop-2, anti-E-cadherin and anti-galectin-3 antibodies as described previously (23).

When an intracellular antigen was detected, cells were fixed with methanol at −20 °C for 40 min, and then washed with PBS. After permeabilization with PBS containing 1% TX-100 at room temperature for 20 min, the cells were treated with methanol at −20 °C for 40 min. After blocking with PBS containing 5% BSA and 0.1% TX-100, the cells were incubated with primary antibodies (rabbit anti-HA tag and mouse anti-β-catenin antibodies), and then stained with fluorescence-conjugated secondary antibodies and DAPI. Finally, the stained cells were observed under a confocal laser-scanning fluorescence microscope (Leica) at a magnification of ×630.

### Immunohistochemistry

Immunohistochemical staining was performed as described previously (54). Briefly, sections of paraffin-embedded tumor tissues were de-paraffinized in xylene and then de-hydrated through graded ethanol series. The sections were heated for 10 min at 105 °C in an autoclave in Target Retrieval Solution (DAKO). The sections were then incubated with 3% hydrogen peroxide to block endogenous peroxidase activity. The specimens were incubated with rabbit anti-phosphorylated Trop-2 antibodies, goat anti-Trop-2 antibodies, and mouse anti-E-cadherin antibodies, and then successively with each HRP-conjugated secondary antibody. Specimens of gastric cancer tissues (20 cases) were obtained from cancer patients in accordance with a protocol approved by the Osaka City University Ethics Committee (approval number 924). Written informed consent for research was obtained from the patients. All human aspects of this study abide by the Declaration of Helsinki principles.

### Treatment with inhibitors

Cells were treated with β-catenin inhibitors (20 μM XAV939, Cayman Chemical) or (100 μM PNU74654, Enzo Life Science) for 24 h, and with PKC inhibitor (1 μM Gö6983, Sigma-Aldrich) or DMSO for the indicated times.

### Treatment with phorbol 12-myristate 13-acetate (PMA)

Cells were treated with 100 ng/ml PMA or its analog, 4α-PMA, for the indicated times.

### Treatment with siRNAs

siRNAs of β-catenin, TCF4, STAT3 and control siRNA (Silencer Select Negative Control #1) were purchased from Ambion. Treatment of cells with siRNAs of β-catenin (5 nM), TCF4 (5 nM), or STAT3 (15 nM), and control siRNA was performed as described previously (23). The sequences of the siRNAs were as follows: β-catenin, 5′-CUGUUGGAUUGAUUCGAAAtt-3′ and 5′-UUUCGAAUCAAUCCAACAGta-3′; TCF4, 5′-GAUGGAAGCUUACUAGAUUtt-3′ and 5′-AAUCUAGUAAGCUUCCAUCtg-3′; STAT3, 5′-GCCUCAAGAUUGACCUAGAtt-3′ and 5′-UCUAGGUCAAUCUUGAGGCct-3′.

siRNAs of Trop-2 and its control siRNA (Control siRNA-A) were purchased from Santa Cruz Biotechnology. Treatment of the cells with three to five Trop-2 target-specific 19 to 25 nt siRNAs and control siRNAs was performed according to the manufacturer’s instructions.

### Construction of a luciferase vector and dual luciferase assays

A luciferase construct was prepared basically as described previously (55). Briefly, the promoter region of ZEB1 (−917∼+56) was amplified from the genomic DNA of HCT116 cells using the following primers: 5′-ATA GGT ACC GCC TGT GGA TAC CTT AGC TC-3′ and 5′-CTG AAG CTT CGC TTG TGT CTA AAT GCT CG -3′. After cloning and digestion with KpnI/HindIII, the fragments were introduced into PicaGene Basic Vector 2 (Toyo Ink: ZEB1 vector).

Dual luciferase assays were performed using an empty vector, the ZEB1 vector, and the pRL-TK vector (Tokyo Ink) as described previously (55).

### Binding of galectin-3 to Trop-2

Galectin-3 bound Sepharose was added to the lysates of WT cells, MCF-cells, and DU145 cells, followed by incubation at 4 °C for 3 h in the presence or absence of 50 mM lactose. After washing with PBS, proteins bound to beads were subjected to SDS-PAGE, followed by immunoblotting and detection of Trop-2.

### Treatment with galectin-3

After washing WT cells, MCF-7 cells, and DU145 cells with serum-free DMEM containing 50 mM lactose and serum-free DMEM successively, the cells were treated with 40∼80 μg/ml of galectin-3 in the serum-free medium for the indicated times.

### Migration assay

Migration assay was performed as described previously (23). Briefly, WT-HA cells (4 × 10^4^ cells) treated with or without galectin-3 were suspended in serum-free medium containing 0.2% BSA and then seeded into the upper chamber of a Transwell (24-well culture plate, pore size 8.0 μm; Corning Inc) precoated with fibronectin. After filling the lower wells with serum-containing medium, the cells were incubated for 20 h. The chambers were fixed with methanol and then stained with hematoxylin. The migration was quantified by counting the migrated cells in five randomly selected fields at a magnification of ×100.

### Detection of FLAG-tagged Trop-2 in lung and liver

Cells (5 × 10^6^ cells) were injected into 6-week-old BALB/c nu/nu male mice intraperitoneally and subcutaneously, and at 4 and 8 weeks after inoculation, respectively, lungs and livers were harvested, and used for biochemical detection of FLAG-tagged Trop-2. When the effect of Gö6983 was examined, nude mice intraperitoneally inoculated with WT cells as described above were treated daily by intraperitoneal injection of Gö6983 (0.25 mg/mouse/day). Mice were handled in accordance with the guidelines of the animal committee of Kyoto Sangyo University.

### RT-qPCR

Extraction of total RNAs and RT-qPCR were performed using TRIzol reagent and QuantStudio3 Flex Real-time PCR system (Thermo Fisher Scientific), respectively. Gene specific primers for E-cadherin (One step TB Green PrimeScript RT-PCR Kit II) were obtained from Takara Bio. The PCR conditions for gene amplification were as follows: one cycle (42 °C for 5 min and 95 °C for 10 s) and 40 cycles (95 °C for 5 s and 60 °C for 30 s). Data were normalized with GAPDH. The sequences of the primers used were E-cadherin: 5′-GTCACTGACACCAACGATAATCCT-3′ (sense) and 5′- TTTCAGTGTGGTGATTACGACGTTA-3′ (antisense); GAPDH: 5′- TGCACCACCAACTGCTTAG-3′ (sense) and 5′- GGATGCAGTGATGATGTTC-3′ (antisense).

### Statistical analysis

Differences between two groups were analyzed using Student’s *t* test. Differences between three or more groups were assessed by ANOVA, followed by a Tukey’s or Dunnett’s test. All statistical analyses were conducted with SPSS Statistics 25 (IBM).

## Data availability

All data are contained within the manuscript.

## Supporting information

This article contains supporting information.

## Conflict of interest

The authors declare that they have no known competing financial interests or personal relationships that could have appeared to influence the work reported in this paper.

## Supporting information

Supporting Figure S1 **Association of β-catenin with Trop-2 in four cell types.** FLAG-Trop-2 was immunoprecipitated from lysates of WT, SA and SE cells. FLAG-tagged Trop-2 and co-immunoprecipitated β-catenin were subjected to SDS-PAGE, followed by immunoblotting and detection of FLAG-tagged Trop-2 and β-catenin. The densities of bands were determined, and the ratio of β-catenin to FLAG-tagged Trop-2 was calculated. That in WT cells was taken as 1 (means ± S.E., n = 3, n.s.: not significant).

Supporting Figure S2 **Effect of PMA on expression of ZEB1, Twist1 and E-cadherin.***A*, WT-HA cells were treated with PMA (100 ng/ml) or 4α-PMA for 24 h, and cell lysates were subjected to SDS-PAGE and immunoblotting, followed by detection of ZEB1, Twist1, E-cadherin and β-actin. The densities of bands were determined, and the ratios of ZEB1 (*B*), Twist1 (*C*), and E-cadherin (*D*) to β-actin were calculated. The value in WT-HA cells treated with 4α-PMA was taken as 1 (means ± S.E., n = 3, ∗∗*p* < 0.01, n.s.: not significant).

Supporting Figure S3 **Effect of growth factors on Trop-2 cleavage in WT-HA cells.***A*, the levels of growth factor receptor mRNA in WT cells were determined by DNA microarray analysis (n = 1). *B*, WT-HA cells were treated with EGF, FGF, HGF, IGF-1 (10 ng/ml) and TGF-β (100 ng/ml) for 1 h. HA-tagged Trop-2 was immunoprecipitated from lysates and subjected to SDS-PAGE and immunoblotting. The ratio of HA-tagged ∼13 kDa fragment to HA-tagged intact Trop-2 was analyzed as in Figure 3*B* (means ± S.E., n = 3, n.s.: not significant).

Supporting Figure S4 **Effect of galectin-3 on the expression of ZEB1 and E-cadherin in SA cells.** SA cells were treated with or without galectin-3 (80 μg/ml) for 24 h, and then cell lysates were subjected to SDS-PAGE and immunoblotting, followed by detection of ZEB1, E-cadherin and β-actin. The densities of the bands were determined, and the ratio of ZEB1 and E-cadherin to β-actin was calculated. That in galectin-3-non-treated cells was taken as 1. β-Actin served as a loading control (means ± S.E., n = 3, n.s.: not significant).

Supporting Figure S5 **Cell morphology of four cell types cultured for 7 days.***A*, photomicrographs of four cell types (Mock, WT, SA and SE cells) were taken after culture for 7 days. Scale bars, 100 μm. *B*, lysates of WT cells cultured for 1 and 7 days were subjected to SDS-PAGE and immunoblotting, followed by detection of E-cadherin and β-actin. The densities of bands were determined, and the ratio of E-cadherin to β-actin was calculated. The value in WT cells cultured for 1 day was taken as 1 (means ± S.E., n = 3, ∗*p* < 0.05). *C*, cell surface galectin-3 of WT cells cultured for 1 and 7 days was observed as described in Figure 6*B*. Scale bars, 10 μm.

Supporting Figure S6 **Expression of Trop-2 in MCF-7 cells and DU145 cells.***A*, Trop-2 was immunoprecipitated from lysates of MCF-7 cells and DU145 cells, and then subjected to SDS-PAGE and immunoblotting, followed by detection with anti-Trop-2 antibodies. *B*, expression of Trop-2 in MCF-7 cells and DU145 cells was analyzed by flow cytometry (Anti-Trop-2 antibody, solid line; control IgG, *gray shading*).

Supporting Figure S7 **Expression of N-cadherin and Vimentin in four cell types.***A*, the levels of N-cadherin and Vimentin mRNAs in four cell types were determined by DNA microarray analysis (n = 1), and that in Mock cells was taken as 1. *B*, the levels of N-cadherin and Vimentin proteins were determined as described Figure 1*D*. β-Actin served as a loading control (means ± S.E., n = 3, ∗*p* < 0.05, ∗∗*p* < 0.01, n.s.: not significant).

Supporting Figure S8 **Enhancement of E-cadherin mRNA in SE cells treated with β-catenin siRNA.** SE cells were treated with β-catenin siRNA and control siRNA as described in Figure 4. The levels of E-cadherin mRNA were determined by qRT-PCR, and those in SE cells treated with control siRNA were taken as 1 (means ± S.E., n = 3, ∗∗*p* < 0.01).
