# Supplementary figures and images for: Trophoblast cell surface antigen-2 phosphorylation triggered by binding of galectin-3 drives metastasis through down-regulation of E-cadherin
Source: J Biol Chem. 2023 Jun 27;299(8):104971. doi: 10.1016/j.jbc.2023.104971 (PMC10392139; doi:10.1016/j.jbc.2023.104971)

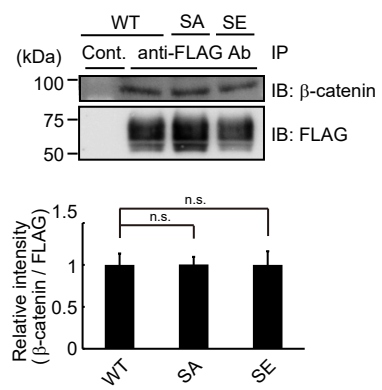

Supplementary Figure S1

Supplement: Supporting Figure S1 — Association of β-catenin with Trop-2 in four cell types. FLAG-Trop-2 was immunoprecipitated from lysates of WT, SA and SE cells. FLAG-tagged Trop-2 and co-immunoprecipitated β-catenin were subjected to SDS-PAGE, followed by immunoblotting and detection of FLAG-tagged Trop-2 and β-catenin. The densities of bands were determined, and the ratio of β-catenin to FLAG-tagged Trop-2 was calculated. That in WT cells was taken as 1 (means ± S.E., n = 3, n.s.: not significant). [file mmc1.pdf]

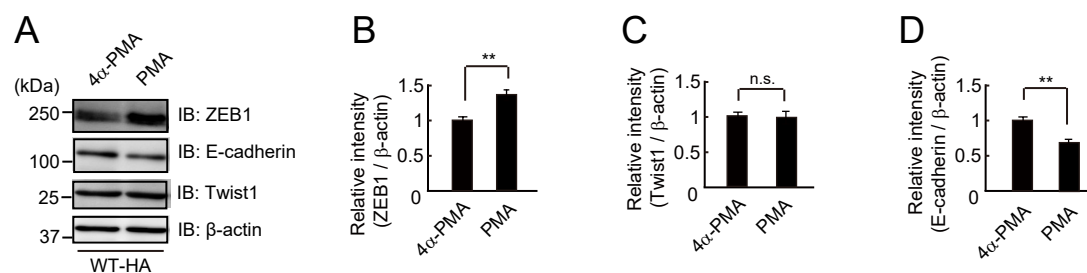

Supplementary Figure S2

Supplement: Supporting Figure S2 — Effect of PMA on expression of ZEB1, Twist1 and E-cadherin.A, WT-HA cells were treated with PMA (100 ng/ml) or 4α-PMA for 24 h, and cell lysates were subjected to SDS-PAGE and immunoblotting, followed by detection of ZEB1, Twist1, E-cadherin and β-actin. The densities of bands were determined, and the ratios of ZEB1 (B), Twist1 (C), and E-cadherin (D) to β-actin were calculated. The value in WT-HA cells treated with 4α-PMA was taken as 1 (means ± S.E., n = 3, ∗∗p < 0.01, n.s.: not significant). [file mmc2.pdf]

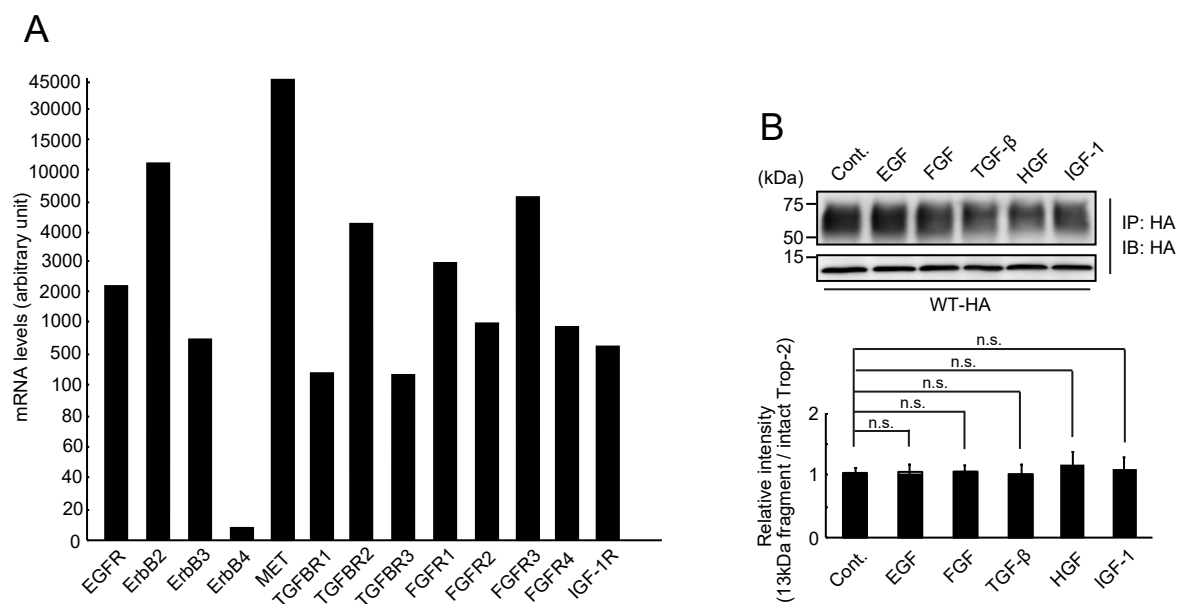

Supplementary Figure S3

Supplement: Supporting Figure S3 — Effect of growth factors on Trop-2 cleavage in WT-HA cells.A, the levels of growth factor receptor mRNA in WT cells were determined by DNA microarray analysis (n = 1). B, WT-HA cells were treated with EGF, FGF, HGF, IGF-1 (10 ng/ml) and TGF-β (100 ng/ml) for 1 h. HA-tagged Trop-2 was immunoprecipitated from lysates and subjected to SDS-PAGE and immunoblotting. The ratio of HA-tagged ∼13 kDa fragment to HA-tagged intact Trop-2 was analyzed as in Figure 3B (means ± S.E., n = 3, n.s.: not significant). [file mmc3.pdf]

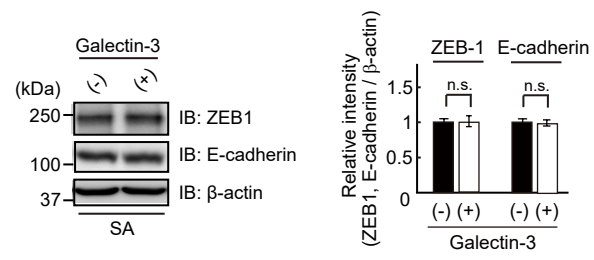

Supplementary Figure S4

Supplement: Supporting Figure S4 — Effect of galectin-3 on the expression of ZEB1 and E-cadherin in SA cells. SA cells were treated with or without galectin-3 (80 μg/ml) for 24 h, and then cell lysates were subjected to SDS-PAGE and immunoblotting, followed by detection of ZEB1, E-cadherin and β-actin. The densities of the bands were determined, and the ratio of ZEB1 and E-cadherin to β-actin was calculated. That in galectin-3-non-treated cells was taken as 1. β-Actin served as a loading control (means ± S.E., n = 3, n.s.: not significant). [file mmc4.pdf]

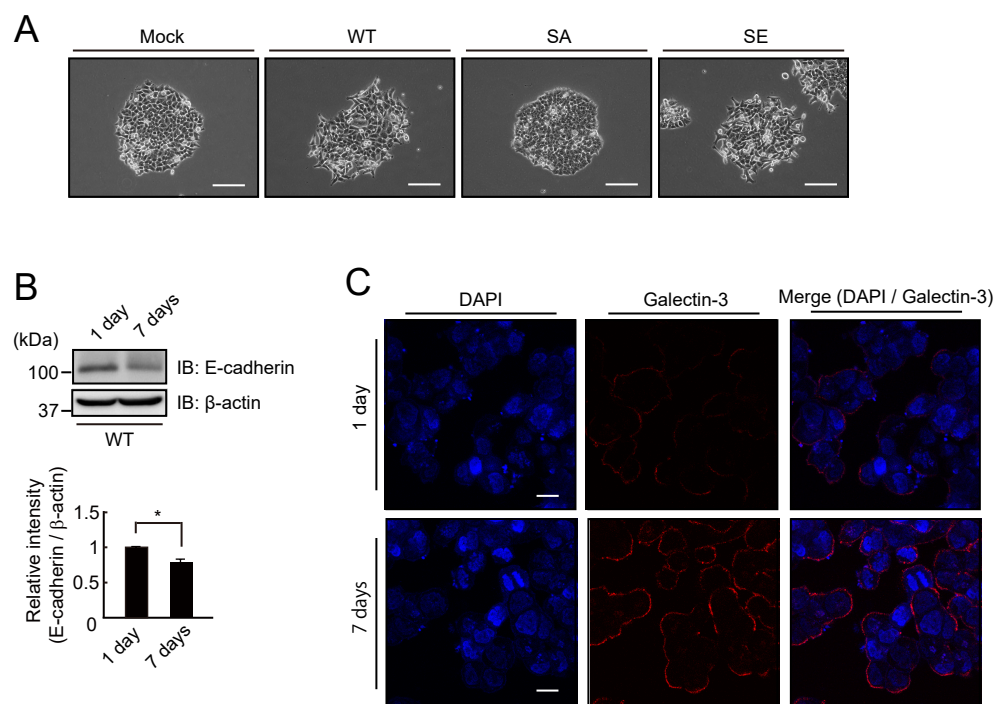

Supplementary Figure S5

Supplement: Supporting Figure S5 — Cell morphology of four cell types cultured for 7 days.A, photomicrographs of four cell types (Mock, WT, SA and SE cells) were taken after culture for 7 days. Scale bars, 100 μm. B, lysates of WT cells cultured for 1 and 7 days were subjected to SDS-PAGE and immunoblotting, followed by detection of E-cadherin and β-actin. The densities of bands were determined, and the ratio of E-cadherin to β-actin was calculated. The value in WT cells cultured for 1 day was taken as 1 (means ± S.E., n = 3, ∗p < 0.05). C, cell surface galectin-3 of WT cells cultured for 1 and 7 days was observed as described in Figure 6B. Scale bars, 10 μm. [file mmc5.pdf]

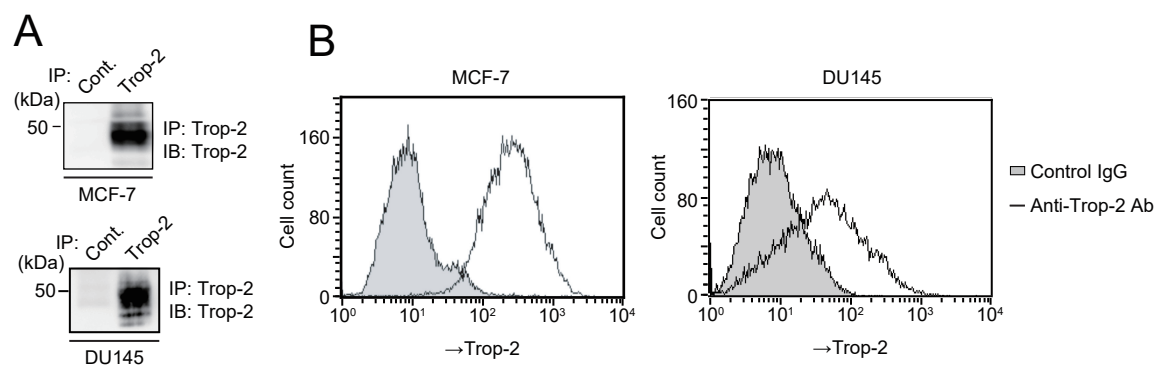

Supplementary Figure S6

Supplement: Supporting Figure S6 — Expression of Trop-2 in MCF-7 cells and DU145 cells.A, Trop-2 was immunoprecipitated from lysates of MCF-7 cells and DU145 cells, and then subjected to SDS-PAGE and immunoblotting, followed by detection with anti-Trop-2 antibodies. B, expression of Trop-2 in MCF-7 cells and DU145 cells was analyzed by flow cytometry (Anti-Trop-2 antibody, solid line; control IgG, gray shading). [file mmc6.pdf]

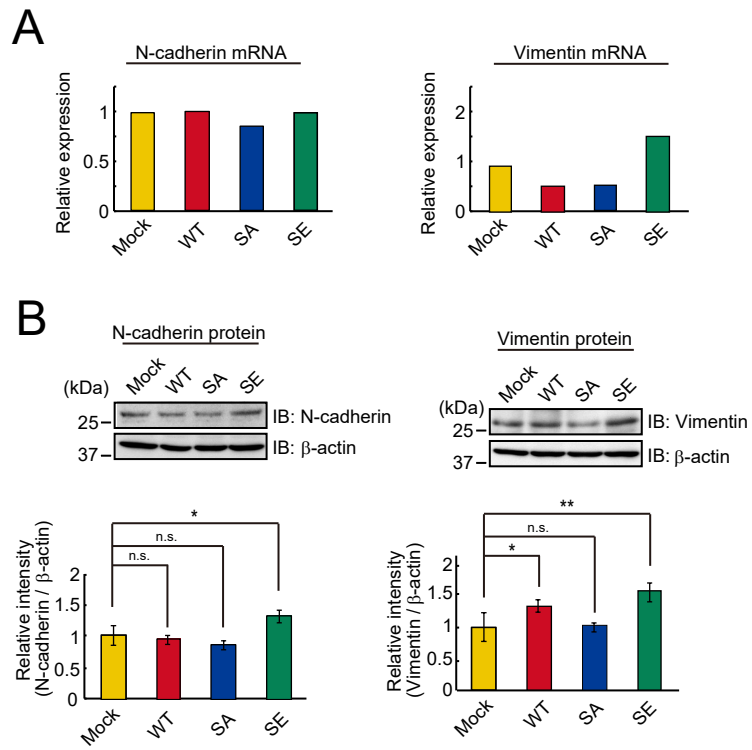

Supplementary Figure S7

Supplement: Supporting Figure S7 — Expression of N-cadherin and Vimentin in four cell types.A, the levels of N-cadherin and Vimentin mRNAs in four cell types were determined by DNA microarray analysis (n = 1), and that in Mock cells was taken as 1. B, the levels of N-cadherin and Vimentin proteins were determined as described Figure 1D. β-Actin served as a loading control (means ± S.E., n = 3, ∗p < 0.05, ∗∗p < 0.01, n.s.: not significant). [file mmc7.pdf]

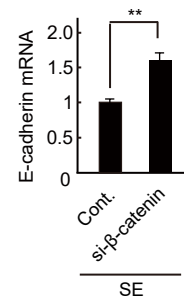

Supplementary Figure S8

Supplement: Supporting Figure S8 — Enhancement of E-cadherin mRNA in SE cells treated with β-catenin siRNA. SE cells were treated with β-catenin siRNA and control siRNA as described in Figure 4. The levels of E-cadherin mRNA were determined by qRT-PCR, and those in SE cells treated with control siRNA were taken as 1 (means ± S.E., n = 3, ∗∗p < 0.01). [file mmc8.pdf]
